# Antibiotic Therapy of Plague: A Review

**DOI:** 10.3390/biom11050724

**Published:** 2021-05-12

**Authors:** Florent Sebbane, Nadine Lemaître

**Affiliations:** 1 Univ. Lille, Inserm, CNRS, Institut Pasteur Lille, U1019—UMR 9017—CIIL—Center for Infection and Immunity of Lille, F-59000 Lille, France; 2 Laboratoire de Bactériologie-Hygiène, Centre Hospitalier Universitaire Amiens Picardie, UR 4294, Agents Infectieux, Résistance et Chimiothérapie (AGIR), Université de Picardie Jules Verne, F-80000 Amiens, France

**Keywords:** plague, antimicrobial chemotherapy, multidrug resistance, anti-virulence drugs, phage, immunotherapy, predatory bacteria, host-directed therapies, vaccine strain, nutritional immunity

## Abstract

Plague—a deadly disease caused by the bacterium *Yersinia pestis*—is still an international public health concern. There are three main clinical forms: bubonic plague, septicemic plague, and pulmonary plague. In all three forms, the symptoms appear suddenly and progress very rapidly. Early antibiotic therapy is essential for countering the disease. Several classes of antibiotics (e.g., tetracyclines, fluoroquinolones, aminoglycosides, sulfonamides, chloramphenicol, rifamycin, and β-lactams) are active in vitro against the majority of *Y. pestis* strains and have demonstrated efficacy in various animal models. However, some discrepancies have been reported. Hence, health authorities have approved and recommended several drugs for prophylactic or curative use. Only monotherapy is currently recommended; combination therapy has not shown any benefits in preclinical studies or case reports. Concerns about the emergence of multidrug-resistant strains of *Y. pestis* have led to the development of new classes of antibiotics and other therapeutics (e.g., LpxC inhibitors, cationic peptides, antivirulence drugs, predatory bacteria, phages, immunotherapy, host-directed therapy, and nutritional immunity). It is difficult to know which of the currently available treatments or therapeutics in development will be most effective for a given form of plague. This is due to the lack of standardization in preclinical studies, conflicting data from case reports, and the small number of clinical trials performed to date.

## 1. Introduction

Plague is one of the most infamous and most feared diseases because it has caused three pandemics, with consequential disruption of the political, social, economic, cultural and religious orders [1,2,3,4,5,6,7,8]. It has been estimated that plague caused more than 150 million deaths across the world. However, the number of plague cases has decreased over time. Plague killed hundreds of Europeans in the early 20th century, while it killed millions in the Middle Ages. In France, the last known case was recorded in 1945. It is therefore not surprising that many Europeans consider plague to be an ancient and extinct disease. However, this is not the case; 11 countries in sub-Saharan Africa, Asia, and the Americas reported around 3000 human cases of plague to the World Health Organization (WHO) between 2013 and 2018 [9]. Of these, Madagascar and the Democratic Republic of Congo accounted respectively for ~80% and ~15% of the world’s cases of plague.

It must be borne in mind that plague mainly circulates between rodents and their fleas; ~200 species of rodents and lagomorphs and ~80 fleas have been associated with plague, but few are considered significant hosts or vectors (see [10] for a brief overview of plague epidemiology). The geographical distribution of outbreaks in the animal reservoir differs from that of outbreaks in humans. Of the 33 countries that contained a known animal plague focus at some time in the last 30 years, only 10 reported human cases between 2013 and 2018 [9]. Hence, the re-emergence of human plague after decades of silence in certain areas where plague is considered extinct may indicate the presence of an ancient animal reservoir that is active but relatively isolated from the human population. This is one possible explanation for the re-emergence of human plague in Algeria and Libya after an absence of 20 to 50 years [11,12,13]. In any case, the animal surveillance data and the occurrence of human cases in previously disease-free areas suggest that the territories occupied by plague are growing. In addition to this natural threat, the use of the plague bacillus by individuals or states for warfare or terrorism (as has already occurred in the past) is also of great concern [14]. Lastly, the threat of plague is compounded by the emergence of strains resistant to the antibiotics of choice for disease control or the potential use with harmful intent of strains that have deliberately been rendered resistant to our entire therapeutic arsenal [15,16]. For this reason, several research groups are developing antiplague strategies. Some of these therapeutic strategies are broad-spectrum, and others are plague-specific. Here, we review the literature data on the current treatment of plague and the therapeutic strategies in development. However, we will first briefly describe the main clinical forms of plague.

## 2. The Different Clinical Forms of Plague

Bubonic plague, septicemic plague and pneumonic plague are the three main clinical forms of this disease. Bubonic plague accounts for 70% to 90% of human cases, whereas the septicemic and pneumonic forms respectively account for around 30% and less than 5% [17,18,19,20,21,22]. It is noteworthy that the septicemic form is mainly diagnosed in USA because of the increasing standardized blood culture programs implemented in the country’s hospital laboratories [23]. The incubation period for plague is usually 2 to 3 days, although periods of 1 or even 5 days are not uncommon [17,24]. Regardless of the form of plague, the symptoms (fever, headache, general malaise, etc.) appear suddenly in most cases [17,24]. Furthermore, the disease progression is very rapid and usually fatal. However, the death rate is much lower for bubonic plague (40–60%) than for septicemic or pneumonic plague (>90% in both cases) [17,23]. Less commonly diagnosed clinical manifestations of plague included pestis minor (a benign form of bubonic plague), carbuncular plague with or without palpable buboes, gastric plague, and meningitis [25,26,27,28,29]. These minor forms of disease may reflect disease caused by atypical strains of *Y. pestis* or disease progression in immunocompromised patients and/or patients who receive inappropriate treatment [30,31,32].

Bubonic plague is the oldest known form of the disease, as depicted in paintings of Saint Roch showing a characteristic bubo on his groin. Although most buboes are located in the inguinal lymph nodes, they can also occur in the axilla (in 20% of cases) and most rarely in the neck (5%). The bubo appears after *Y. pestis* has been inoculated into skin, usually after the bite of an infected flea [33]. At a certain point in the lymph node infection, the bacterium escapes into the bloodstream [34]. Active replication in the circulation produces severe bacteremia, so-called secondary septicemic plague since it occurs after colonization of the lymph node. At the terminal stage of infection, all the organs are heavily colonized. The patient or animal is ultimately killed by disseminated intravascular coagulation.

Primary septicemic plague is characterized by a fatal systemic infection in the absence of bubo production. Hence, the clinical picture is nonspecific, with general organ system failure. Histological and bacteriological analyses have revealed that 10% to 30% of mice bitten by infected fleas succumb to fatal bacteremia without developing a bubo [21,31,32,35]. Thus, the flea transmits both bubonic plague and primary septicemic plague. The more common bubonic plague results from the regurgitation of bacteria into the extravascular part of the dermis, whereas the less frequent primary septicemic plague results from the regurgitation of bacteria directly into the vascular part of the dermis and does not require the additional factors involved in the production of bubonic plague.

A patient’s lungs may be colonized by *Y. pestis* disseminated in the blood. In 5% of cases, this colonization produces secondary pneumonic plague, i.e., plague in which septicemia is followed by pneumonia [17,24]. In a clinical examination, the patient has intense fever, cough, and chest pain when breathing, due to inflammation of the pleura (pleurisy). The inflammatory foci are located in the middle lobe of the right lung or in the upper lobes of both lungs. This characteristic pattern should prompt the physician to suspect pneumonia of septicemic origin [36]. In most cases, the patient coughs up sputum with increasing frequency and (before death) coughs up blood. Patients with secondary pneumonic plague can also infect other people by breathing out *Y. pestis*-containing aerosols. In this case, colonization of the lungs leads to sepsis and therefore deep organ colonization. However, experiments in animal models suggest that systemic infection is not the cause of death. In fact, the combination of rapid bacterial colonization, an intense inflammatory response by the host and then exacerbation of this inflammatory response by *Y. pestis* destroys the lung’s architecture and induces edema and hemorrhage. This pneumonia (referred to as primary pneumonic plague) is fatal and highly contagious [37,38,39,40]. Clinically, this form is characterized by the sudden onset of fever, severe headache, and vomiting, followed by chest pain, shortness of breath, and delirium. The patient eventually falls into a coma and dies [41].

## 3. Antimicrobial Chemotherapy

The premises of antibiotic therapy of infectious diseases go back a long way [42]. However, the idea of chemotherapy using purified or synthesized molecules only emerged just over a century ago (in 1911), when arsphenamine was used to treat syphilis. After this discovery and that of penicillin in 1928 [43], around 15 classes of antibiotics with different modes of action were described between 1932 and 1987; the 1940s, 1950s, and 1960s constituted the golden age of antibiotic discovery [44]. Although known compounds have been improved since then, the most recent class of antibiotic with activity against Gram-negative bacilli was introduced in the late 1980s [44].

It is undeniable that the advent of antibiotics has greatly reduced the death rate for plague patients [22,26,45,46]. However, early administration remains essential. Even when antibiotics known to be effective are given, patients who develop the bubonic form of plague are most likely to survive: after treatment with antibiotics, around 10% of patients with bubonic plague and 30% to 50% of patients with pneumonic or septicemic plague will nevertheless die [22,23,45,47]. However, a recent meta-analysis of deaths associated with pneumonic plaque and data from the large outbreak of pneumonic plague in Madagascar in 2017 indicated that the death rate in patients with confirmed or probable pneumonic plague was between 8–25% [48,49].

### 3.1. Data from In Vitro Experiments

In vitro, measurement of the minimum inhibitory concentration (MIC) in a microdilution assay that complied with the Clinical and Laboratory Standards Institute’s guidelines for the *Enterobacteriaceae* revealed that tetracyclines, fluoroquinolones, aminoglycosides, sulfonamides, and most of β-lactams are active against *Y. pestis*; the MIC_90_ ranged from below 0.125 mg/L to 4 mg/L (Table 1) [50,51,52,53,54,55,56,57,58,59,60,61,62,63,64,65,66,67,68,69,70,71]. However, few studies have determined the MICs in more than one strain, and most of the MIC assays were performed at *Yersinia* optimal growth temperature (28 °C)—a temperature at which the lipooligosaccharide’s structure is not the same as at 37 °C [72]. Consequently, antibiotics that are efficacious at 28 °C might not be at 37 °C (and inversely) due to a change in membrane fluidity (as observed for polymyxin) [72]. Along with in vitro MIC studies, the in vitro hollow-fiber pharmacodynamic model can be used to simulate clinical regimens [62,65]. In some respects, this model is better than small animal models for assess the true efficacy of certain drugs. Indeed, drug half-lives in small animals are often shorter than those in humans. The results of hollow-fiber studies suggest that ampicillin, meropenem, ciprofloxacin, moxifloxacin, and gentamicin are more efficacious than streptomycin [73]. The relatively low efficacy of streptomycin is related to the easy acquisition of resistance by mutants in this in vitro model, rather than to poor activity of the drug per se. Despite some advantages, the hollow-fiber model prevents a full evaluation of the available panel of antibiotics, some of which are recommended by the US Food and Drug Administration for the treatment of plague. Firstly, the hollow-fiber model does not include immune system components able to eliminate bacteria weakened by exposure to bacteriostats like doxycycline [63]. Secondly, this model cannot take into account certain pathophysiological aspects (such as necrosis and the purulent aspect of infected tissues) that limit the action of certain antibiotics. For instance, experiments with the hollow-fiber model suggest that gentamicin and ciprofloxacin should be similarly effective in the treatment of *Y. pestis* infections in humans [73]. However, although ciprofloxacin protects mice from bubonic plague as well as gentamicin does, only ciprofloxacin eradicates *Y. pestis* from the lymph nodes and thus avoids the risk of relapse after treatment cessation [74]. Taken as a whole, these data emphasize the limitations of the hollow-fiber model and the need to evaluate drugs in animal models.

### 3.2. Data from Animal Models

The efficacy of aminoglycosides, tetracyclines, fluoroquinolones, β-lactams, rifamycin, chloramphenicol, sulfonamides and ketolides has been evaluated in rodent (rat and mouse) models of plague and (less frequently) in non-human primate models of pneumonic plague (Table 2) [17,52,56,61,63,69,75,76,77,78,79,80,81,82,83,84,85,86,87,88,89,90,91,92,93,94,95,96,97,98,99,100,101,102,103,104,105,106,107,108,109,110,111,112]. The resulting degree of protection against plague often varies from one animal study to another. These discrepancies might be due to interstudy differences in the *Y. pestis* strain, the bacterial growth conditions (known to impact gene expression), the animal species, the animal strain, the rearing conditions (known to influence the immune response), the inoculation dose, the antibiotic administration route, the time interval between *Y. pestis* inoculation and antibiotic administration, and the duration of treatment (Table 2). For instance, a treatment based on doxycycline, ampicillin or cefoperazone could be recommended on the basis of data obtained with the *Y. pestis* strains typically used in animal models and which are characterized by the production of a protein capsule (F1). However, treatment with any of these three antibiotics is less efficacious in mice infected with a strain that lacks an F1 capsule [78,82,83,113]. This information is particularly important because the F1 capsule is not essential for virulence and some natural isolates lack the F1 capsule [31,78,82,83,114,115,116,117,118]. Similarly conflicting results have been observed with the β-lactams. Third-generation cephalosporins (ceftriaxone) were not efficacious in a mouse model of pneumonic plague [56]. A lack of in vivo efficacy of ceftriaxone and carbapenems was also reported in a mouse model of septicemic plague [89]. In contrast, Bonacorsi et al. came to the opposite conclusion in a study of a mouse model of septicemic plague. The researchers found that β-lactams such as cefotaxime (another third-generation cephalosporin with the same bacterial spectrum of action as ceftriaxone) were as bactericidal in vivo as fluoroquinolones and aminoglycosides [52]. These three studies used different *Y. pestis* strains, mouse strains, administration routes for inoculation and antibiotics, inoculation doses, and times to antimicrobial drug initiation. Given the observed variability, it would be advisable to set up a standardized protocol based on relevant criteria. However, even with the best standards and animal models, predicting the efficacy of one treatment vs. another is complicated by the fact that drug half-lives are often shorter in small animals than in humans [119], and there are few studies of non-human primates. In other words, data from animal models can only indicate potential efficacy in humans; clinical studies are required to establish the true efficacy of one treatment vs. another. However, clinical studies in this field are very scarce.

### 3.3. Treatment Administration Modes

Antibiotics can be given orally, subcutaneously, intravenously or by inhalation. In cases of plague, the choice of the administration route is influenced by the patient’s clinical profile and the form of the disease. The dermal route is not recommended, whereas the intravenous (IV) route is widely recommended for patients with late-stage disease. Indeed, IV injection circumvents the barriers encountered during oral or subcutaneous administration and allows faster delivery of the molecules to the infected tissues; this rapid action is essential for the treatment of a fast-progressing disease like plague. However, IV administration is affected by clearance mechanisms, and the drug is diluted in the circulation. As a result, only a small proportion of the injected antibiotic molecules reaches the lungs. This is why the value of inhaled antibiotics in the treatment of respiratory diseases has been investigated; lower total doses of antibiotics can be used to achieve high local concentrations in the lungs, reduce systemic exposure and thus diminish the risk of adverse events. Following the marketing authorization of inhaled tobramycin in 1997, this approach was used to successfully treat patients with cystic fibrosis infected with *Pseudomonas aeruginosa* [120]. A comparative study of mice intranasally infected with pulmonary plague showed that inhalation of an aminoglycoside antibiotic (gentamicin) was more efficacious than subcutaneous injection [110]. Interestingly, the researchers results suggested that although subcutaneously injected antibiotics reached and protected deep organs, the drugs did not necessarily reach the lungs (depending on differences in diffusion properties, e.g., ciprofloxacin vs. gentamicin): hence, protection in the lungs might be mediated solely by the host’s immune system. Elimination of bacteria from the lungs takes much longer—perhaps because the quantities of antibiotic reaching these organs are too low. In fact, a combination of inhaled and injected antibiotic treatment by subcutaneous route rapidly eliminated the bacteria from all the infected organs [110]. The superiority of the latter treatment (a combination of inhalation and subcutaneous inoculation) over 24 h/24 h intravenous injection is questionable. Nevertheless, the combination can be administered more easily by most medical staff.

### 3.4. Data from Clinical Cases and Studies

The clinical data indicate that many classes of antibiotics (i.e., aminoglycosides, tetracyclines, fluoroquinolones, sulfonamides, phenicols, and β-lactams) have been administered to patients with the different forms of plague [22,75,121,122,123]. A review of published case reports found that aminoglycosides, tetracyclines, and fluoroquinolones (but not sulfonamides) were most consistently effective in the treatment of pneumonic plague. However, the higher case fatality rate of pneumonic plague associated with sulfonamides drugs might be due to bias since ancillary care was less effective when the drug was introduced in the 1930s [22,75].

The analysis of case report data showed that patients (i) are not necessarily treated at the same time after infection, (ii) do not necessarily receive the same drug, as a function of their symptoms, and (iii) have different dose levels and dosing frequencies even when the same drug is administered. Furthermore, the exact patient’s clinical background other than plague symptoms (e.g., diabetes, obesity, immunodeficiencies) was often unknown. These differences result in significant bias in selecting the most effective treatment [23] and emphasize the importance of performing randomized clinical trials. Unfortunately, only two comparative clinical studies have been published: one compared sulfonamide drugs with streptomycin in bubonic plaque, and the other compared streptomycin with gentamicin (alone or combined with tetracycline) in all three main clinical forms of plague [75,121]. The data did not indicate that one class of antibiotic was superior to the others. Lastly, in response to Madagascar’s 2017 plague epidemic, a randomized clinical trial of the efficacy of ciprofloxacin alone vs. streptomycin followed by ciprofloxacin is ongoing [122].

Although the scarcity of relevant clinical data makes it difficult to determine the superiority of one treatment over another [22,121,123,124], the antibiotics’ pharmacodynamic and pharmacokinetic properties can be taken into account when selecting treatments. For example, fluoroquinolones might (like tetracyclines) be more efficacious than aminoglycosides because they accumulate in the cell and diffuse more readily into necrotic tissues, such as the bubo [125,126,127]. Consistently, ciprofloxacin (a fluoroquinolone) is more efficacious than gentamicin (aminoglycoside) in killing *Y. pestis* in the lymph nodes of mice with late-stage bubonic plague [74].

Several patients have received combinations of antibiotics [128]. In a few cases, this approach (e.g., a combination of streptomycin and chloramphenicol) was warranted because the patients lived in an area in which a streptomycin-resistant isolate had been documented [128]. In other cases, patients received a combination because the drugs’ potentially additive or synergistic effects might have increased the likelihood of survival. However, most patients received an empirical combination of antimicrobials for the treatment of a life-threatening infection, i.e., prior to the diagnosis of plague. Even though these combinations have been administered to a large number of patients, none appear to be more effective than the other. For example, the survival rate was no greater for a combination of aminoglycosides and tetracycline than for tetracycline alone [22]. It should also be bear in mind that combining antibiotics can increase the incidence of side effects and drug interactions. An accumulation of toxic effects or antagonism within certain antibiotic combinations might have a negative effect and actually worsen the patient’s outcome.

In conclusion, the current guidelines on the treatment of plague are based on preclinical and clinical data (which are not fully reliable) and on the availability of antimicrobial drugs in the country concerned [23,24,129,130]. In the USA and France, streptomycin and tetracycline (recommended by the WHO) have been replaced with gentamicin and doxycycline, respectively [22].

## 4. Antibiotic Resistance

For many years now, various *Y. pestis* strains resistant to the recommended antibiotics have been isolated from both patients and rodents [50,131,132,133,134,135,136]. Genetic studies have indicated that this antibiotic resistance is mediated by three distinct conjugative plasmids (pIP1203, pIP2180H and pIP1202). The pIP1203 and pIP2180H encode monoresistance to streptomycin and doxycycline, respectively [131,132]. The third plasmid (pIP1202) confers resistance to eight antibiotics including all the drugs used for the first-line curative and preventive treatment of plague (streptomycin, tetracycline, chloramphenicol, and sulfonamides) [15,137]. Although the exact origin of these plasmids, their acquisition route and the date of acquisition by *Y. pestis* have not been determined, the source may have been *Enterobacteriaceae* in the flea gut. Indeed, the pIP2180H is homologous to pB71 from *Salmonella enterica* which belongs to the IncH1 group of plasmids. Additionally, the pIP1202 has sequence homology with IncA/C plasmids found in various *Enterobacteriaceae* (*Escherichia. coli*, *Klebsiella* spp., and *Salmonella* spp.) involved in the contamination of farmed meat in the USA as well as in the fish pathogen *Yersinia ruckeri* [137]. Furthermore, pIP1202 transfers readily from *E. coli* to *Y. pestis* in the flea midgut [138]. Therefore, the abundant reservoir of a MDR conjugative plasmid (pIP1202-like) in farm animals and the potential high transfer rate in infected tissues (such as the flea midgut) raise concerns about the possible emergence, spread and maintenance of multidrug-resistant *Y. pestis* strains in endemic areas. The Mongolian *Y. pestis* strain is even more of a cause for concern than a pIP1202-harboring strain because it shows resistance to all currently administered antibiotics other than the fluoroquinolones [16]. However, the exact nature of the multi-resistance mechanism has not been determined. Lastly, one cannot rule out the use of engineered multidrug-resistant (MDR) *Y. pestis* strains in warfare or terrorism. Hence, there is an urgent need for new therapeutic strategies that can protect populations from natural or deliberate infection with *Y. pestis*.

## 5. Potential New Antibiotics and Antivirulence Factors

Historically, the search for antibiotics has focused on drugs that kill bacteria or inhibit their growth in vitro. This strategy led to the production of novel antibiotics with new bacterial targets that are not affected by current resistance mechanisms and which constitute a means of fighting MDR pathogens. Over the last decade, however, a new branch of research has sought to produce molecules that inhibit essential virulence factors in bacterial pathogenesis [139,140,141,142]. Accordingly, these new compounds are referred to as antivirulence drugs.

### 5.1. Novel Antibiotics

#### 5.1.1. LpxC Inhibitors

More than 20 years ago, it was reported that Gram-negative bacterial infections could be countered by targeting enzymes involved in biosynthesis of the bacterial membrane [143]. Consequently, several research groups have engineered molecules that inhibit uridine diphosphate-3-*O*-(R-3-hydroxymyristoyl)-*N*-acetyl-D-glucosamine deacetylase (LpxC), the enzyme that catalyzes the first irreversible step in lipid A biosynthesis [144,145,146,147,148]. Several LpxC inhibitors have been synthesized and were found to be efficacious in vitro against a broad panel of Gram-negative clinical isolates, including several multi-resistant and extremely drug-resistant strains involved in nosocomial infections. Some of the LpxC inhibitors are also active in vitro against *Y. pestis* at low concentrations (MICs ≤0.8 µg/mL) [148,149,150]. Furthermore, the biphenyldiacetylene-based LPC-069 was shown to cure bubonic plague in mice and to be as efficacious as doxycycline [149]. However, the regimen requires to cure the disease was quite drastic: an IV injection of 200 mg/kg LPC-069 every 8 h. In other words, LpxC inhibitors clearly have the potential to treat plague but more potent molecules must be developed and tested, notably against MDR strains. Furthermore, the LpxC inhibitors’ ability to treat pneumonic plague has yet to be tested.

#### 5.1.2. Cationic Antimicrobial Peptides

Cationic antimicrobial peptides of natural origin or synthetic versions based on natural structures have been proposed to treat MDR infections [151]. Synthetic peptides are more active than the natural peptide LL-37 against *Y. pestis* and other bacterial species and so could be considered in the treatment of plague [152]. Furthermore, in vitro studies indicate that a combination of broad-spectrum antimicrobial peptides with antibiotics (tetracycline, minocycline or tigecycline) is effective against *Y. pestis* [153]. However, the therapeutic efficacy of peptides (alone or in combination with other drugs) against plague remains to be proven in appropriate animal models.

### 5.2. Antivirulence Drugs

#### 5.2.1. Drugs Targeting Type Three Secretion Systems and the Yersinia Outer Membrane Proteins

Antivirulence drug development has focused on type 3 secretion systems (T3SSs) and their associated toxins [154]. T3SSs are heteromultimer protein complexes produced by many Gram-negative bacteria, including *Y. pestis* [155,156,157]. In *Y. pestis*, the T3SS exports toxic *Yersinia* outer proteins (Yops) from the bacterium into the phagocyte. Once in the host eukaryotic cell, the Yops interact with various signaling pathways to inhibit both phagocytosis and the development of an appropriate immune response [158,159]. Loss of the T3SS makes the bacterium avirulent, and some Yops have a crucial role in virulence. Therefore, several researchers have looked for drugs that inhibit the expression or assembly of T3SSs or even the translocation of effector proteins into the host cell [155,160,161]. In the case of *Y. pestis*, reduced T3SS expression can result from deregulation or a low copy number of the virulence plasmid (pYV) carrying all genes that encode the T3SS and its exotoxins [155,162]. The screening of a library of tens of thousands of molecules identified less than 10 that inhibited the secretion of *Y. pestis* Yops at a micromolar concentration [163]. This type of screening also detected molecules that directly impacted the T3SSs of enteropathogenic *Yersinia* (*Yersinia pseudotuberculois* and *Yersinia enterocolitica*); these T3SSs are very similar to those of Y. *pestis* [155,162,164]. Lastly, the screening of molecule libraries against T3SSs from other bacterial species also revealed drugs that inhibited *Yersinia*’s T3SSs [165]. These included several classes of inhibitors such as salicylidene acylhydrazides, benzimidazoles, 2,2′-thiobis-(4-methylphenol), (-)-hopeaphenol, acylated hydrazones of various salicylaldehydes, monoanionic squaric acids, α-ketocarboxylic acids, and sulfonamides. Some inhibitors affect the assembly or structure of T3SSs, whereas others modify the expression of loci encoding structural proteins. The screening experiments also revealed compounds (such as *N*-hydroxybenzimidazole) that modify the *lcrF* master regulator of T3SS expression or directly block LcrF’s DNA binding domain [155,166,167,168]. In addition to research targeting the secretory apparatus, other programs are developing inhibitors of the tyrosine kinase YopH or the GTPase-activating protein YopE—both of which that are important for pathogenesis [169,170,171,172,173,174,175,176,177,178]. Although the administration of small-molecule inhibitors of LcrF is associated with a greater survival rate in a mouse model of *Y. pseudotuberculosis* pneumonia [167], it remains to be seen whether the inhibitors specifically targeting T3SS and/or its Yops is an effective treatment for experimental plague.

#### 5.2.2. The Yersiniabactin Iron Acquisition System

Yersiniabactin (Ybt) is a siderophore that allows *Y. pestis* to acquire iron (as Fe^3+^) from the outside environment when the metal is scarce [179,180,181]. This uptake system is essential; in fact, Ybt’s presence is critical for the production of bubonic and pneumonic plague [180,181]. As part of efforts to develop new antimicrobials, it has been suggested that compounds with some of the structural similarities to Ybt might inhibit the growth of *Y. pestis* [182]. Indeed, a dozen synthetic structural analogues have demonstrated a degree of activity against the bacterium. However, only one (a (2E)-2-benzylidene-*N*-hydroxyhydrazine carbo(ox/thio/oximid)-amide derivative) has a MIC low enough to be of interest [183]. In addition to compounds with some of the structural similarities to Ybt, aryl sulfamoyl adenosine derivatives have been synthesized as putative inhibitors of the enzyme YbtE, which is essential in the biosynthesis of Ybt [184,185]. Only the arylic acyl adenylate analogue 59-*O*-[*N*-(salicyl)-sulfamoyl] has been tested against *Y. pestis*; it was found to inhibit bacterial growth in an iron-deficient medium. Although molecules inhibiting the Ybt system might be of value, they appear to be limited to prophylactic use and might not be therapeutically effective in some cases because the Ybt system is not required for colonization of the blood and deep organs [179,180,186,187]. Furthermore, Y. *pestis* spreads rapidly into the blood from the lungs, and the flea regurgitates bacteria directly into the blood in 10 to 30% of cases [32,38].

#### 5.2.3. Inhibition of Cell Adhesion

To produce an infection, *Y. pestis* must attach to host cells. In experiments with human respiratory cell lines, *Y. pestis* attaches to oligosaccharide structures [188,189,190]. Furthermore, the bacterium must be able to attach to phagocytes before injecting its Yops via the T3SS [191]. Therefore, the disruption of *Y. pestis*’ attachment to host cells might have therapeutic value. Several compounds have been found to inhibit adhesion to various respiratory cell lines, and galactosucrose oligosaccharides were found to be the most potent [188,189,190]. Despite these encouraging findings, the approach has not been investigated further.

## 6. Other Innovative Approaches That Might Protect against Plague

### 6.1. The Unexpected Role of the Vaccine Strain EV76 and the F1 Subunit

Surprisingly, the *Y. pestis* vaccine strain EV76 might also have value as a prophylactic treatment [192,193]. Indeed, concomitant injection of EV76 with a virulent strain of *Y. pestis* (either at the same injection site or at two separate sites) confers a degree of protection against both bubonic and pneumonic plague. This prophylaxis might be due to the EV76′s ability to induce “nutritional immunity” to iron (i.e., the host’s limitation of iron’s availability to pathogenic microbes) through the heme- and iron-binding proteins hemopexin and transferrin. However, greater survival is only observed in a bubonic plague model; inoculation with a dose as high as 10^4^ CFUs of EV76 protects more than 60% of the animals from bubonic plague but only delays bacterial colonization in pneumonic plague. Nevertheless, all the mice treated with cephalosporin (ceftriaxone) 48 h after concomitant inoculation of the vaccine strain EV76 and the virulent strain survived. This is especially interesting because although cephalosporin is active in vitro against *Y. pestis* [67], it confers almost no protection against pneumonic plague in vivo [56,193]. It is noteworthy that the F1 antigen (making up the protein capsule) can be administered prophylactically in the same way as EV76 [103]. The highly immunogenic F1 antigen protects mice against bubonic plague when administered within 5 h of infection. It is unlikely that the prophylactic effect observed with EV76 is related solely to the production of its capsule. However, if this is the case, it should be bear in mind that (i) the F1 antigen is not essential for the virulence of *Y. pestis*, and (ii) strains not producing this antigen have been described in the literature and can be selected in animals vaccinated with this antigen [31,114,115,116,117,118].

### 6.2. Predatory Bacteria

Another alternative to antibiotics would be the use of predatory bacteria, i.e., bacteria whose survival necessarily depends on predation of *Y. pestis* [194,195,196]. It has been suggested that the Gram-negative predatory bacteria *Bdellovibrio bacteriovorus* and *Micavibrio aeruginosavorus* can fight bacterial infections—especially those caused by MDR strains. Predatory bacteria have a broad spectrum of action and have been found to be safe in different animal models, with rapid elimination from the tissues regardless of the inoculation route [197,198,199,200,201]. Inoculation with *B. bacteriovorus* might protect against pulmonary plague [195,196]. Indeed, early and repeated intranasal injection of very high doses (10^9^ PFUs) of *B. bacteriovorus* after the intranasal inoculation of *Y. pestis* significantly reduced the load of plague bacillus in the lungs 24 h post-infection [195,196]. Further experiments have shown that pre-exposure then repeated injection of *B. bacteriovorus* after systemic challenge with *Y. pestis* provided some protection in SKH-1 mice [202]. However, such treatment did not protect Balb/c mice in a preliminary study, presumably because SKH-1 and Balb-C mice respond differently to the treatment and/or infection [202]. In other words, the application of predatory bacteria was protective due to active predation and/or their impact on the host’s response to the infection.

### 6.3. Phages and Phage Endolysins

It has been known for more than a century that bacteriophages that infect and sometimes kill bacteria have the potential to treat bacterial infections in vivo [203,204]. More than a dozen phages are known to be active against *Y. pestis* [205]. The first successful phage therapy of plague was reported in 1925; the injection of a phage preparation directly into the bubo saved four sick patients [206]. However, the subsequent treatments were less successful [207,208,209,210]. These discrepancies might have been due to major methodological differences [211,212,213]. Nevertheless, a standardized phage preparation might be a therapeutic alternative to antibiotics. Indeed, intraperitoneal injection of the phage φA1122 delayed death and protected several animals that had received a high dose of *Y. pestis* [205,214]. Although these results are encouraging, it is important to bear in mind that (i) phage resistance is frequent and (ii) phages can carry genetic material that accentuates virulence and antibiotic resistance [215,216]. Hence, phage therapy will necessarily involve the use of a genetically well-defined cocktail of phages.

Phage endolysin (an enzyme that cleaves peptidoglycan) has also been proposed as a therapeutic alternative to the whole phage. However, this approach is problematic for the treatment of Gram-negative bacteria, in which the peptidoglycan is protected by an outer membrane. In order to circumvent this problem, researchers have developed a chimeric fusion protein that enables the phage lysin T4 lysozyme to cross the outer membrane [217,218,219]. This idea was based on the observation that *Y. pestis* produces a muramidase (pesticin) whose bactericidal action involves its passage through the outer membrane of bacteria via the FyuA transporter [220]. Thus, the fusion protein comprises the FuyA-binding region of pesticin and the muramidase region of lysozyme T4. Although this type of chimeric protein might have therapeutic value, its bioavailability in the various organs colonized by *Y. pestis* has not been determined with regard to the administration mode and dose. However, delivery to the lungs might protect against pneumonic plague.

### 6.4. Immunotherapy

Passive immunization has long been used as a means of prophylaxis against infectious diseases. In fact, plague serum was used to control plague more than a century ago [221,222]. The discovery of hybridomas in the early 1970s led to the development of a new class of therapeutic: the monoclonal antibody [223,224,225]. Several monoclonal antibodies have been suggested as plague treatments, including those against virulence factors like the T3SS, Yops [226,227,228,229,230,231,232,233,234,235,236,237] and the F1 capsule [234,238,239]. Indeed, antibodies against the T3SS components (LcrV, YopB, and YopD) inhibit the secretion of Yop exotoxins into the phagocytes and thus restrict Y. *pestis*’ ability to deactivate these immune cells [226,227,228,229,230,231,238]. However, targeting YopB and YopD might only be efficacious against *Y. pestis* strains that do not produce the F1 capsule [231]. Targeting the Yops has been suggested because (i) some Yops are also present on the bacterial cell surface [240], and (ii) one of these, YopE, is a crucial virulence factor. However, antibodies against YopE do not provide satisfactory protection [231]. Lastly, a mixture of polyclonal anti-F1 antibodies and a monoclonal antibody against the lipooligosaccharide or the murine toxin Ymt has also been shown to protect against the disease [241], and anti-F1 and anti-LcrV monoclonal antibodies may be synergistic [232,238]. More importantly, humanized monoclonal anti-F1 and anti-LcrV antibodies protect against bubonic plague in mice [238]. Although the most efficacious monoclonal antibodies (anti-F1 and anti-LcrV) are an attractive alternative to today’s plague treatments, there is a risk of therapeutic failure; F1-negative strains have been described in the literature, the F1 capsule is not essential for plague, and polymorphism in the LcrV antigen is likely [31,114,115,116,117,236,242,243]. Another limitation of antibodies is their low oral bioavailability and the fact they would probably have to be injected intravenously.

### 6.5. Host-Directed Therapies

Recently, a number of anti-infective research programs have focused on the development of molecules targeting host mechanisms/channels that are exploited by the infecting pathogen [244,245,246]. Relative to molecules that target the infectious agent, host-directed therapeutics are much less likely to prompt the development of resistance and so could be used to treat infections by MDR bacteria. It has been suggested that antagonizing the adenosine A1 receptor with the compound L-97-1 can protect against pneumonic plague. The antagonist would prevent the release of immunomodulatory substances that kill endothelial cells after *Y. pestis* lipooligosaccharide interacts with the adenosine A1 receptor and that lead to acute lung injury [247,248,249]. However, L-97-1 was efficacious against pulmonary plague in the rat only when co-administered with an [underdosed] antibiotic [249]. Another immunomodulator (glutoxim) has been tested as a plague prophylactic [41]. However, it only protected half the mice inoculated with a small number of bacteria, and so has not attracted further interest. Among with the molecules identified on the basis of our current hypotheses, other drug candidates have been discovered through the hypothesis-free, high-throughput screening of compound libraries. In one study, three molecules (the phenothiazine antipsychotic trifluoperazine, the respiratory stimulant doxapram, and the tricyclic antidepressant amoxapine) inhibited *Y. pestis*’ ability to kill macrophages, but none was bactericidal, bacteriostatic, or capable of inhibiting the secretion of Yops into the environment [250]. However, the bacterium’s inability to translocate Yops into the host cell was not evaluated. Interestingly, phenothiazine, doxapram, and amoxapine protected 40–60% of the animals in a model of pulmonary plague; however, they did not provide the total protection obtained with a quinolone antibiotic (levofloxacin) [250]. Lastly, prophylaxis with lovastatin gave some protection in a model of primary septicemic plague though an as-yet unidentified host mechanism [101]. It remains to be seen whether this treatment protects against bubonic or pulmonary forms of plague.

### 6.6. Reducing an Excessive Immune Response

Antibiotic therapy affords little protection during the late stage of plague. This is presumably because the host is unable to control a detrimental inflammatory response associated with the rapid dissemination of *Y. pestis* throughout the host. Therefore, inhibiting the inflammatory process might be a means to improve the survival of plague patients. However, this inhibition has to be moderate since the immune response is required for effective bacterial clearance [251]. Levy et al. tested the above idea using a mouse model of bubonic plague [252]. They reported that a mild corticosteroid treatment combined with the administration of anti-*Y. pestis* antibodies had a real beneficial effect, whereas the anti-inflammatory molecule by itself did not confer protection against *Y. pestis*.

## 7. Conclusions

The data obtained in vitro and from animal models, case reports and the few published clinical trials show that several treatments approved by the health authorities have prophylactic or therapeutic activity against plague in humans. Along with our current arsenal of effective antibiotics, new classes of antibiotics and new strategies for plague treatment have emerged in response to the advent of MDR strains of *Y. pestis*. Some of these novel strategies are in late-stage clinical development and appear to have therapeutic value. However, it is difficult to predict which treatments will be most effective for each of the different forms of plague. This is due to the small number of clinical trials that have been conducted and the lack of standardization in animal models. In the future, the use of standardized human tissues and/or microfluidic organoids could be a new way to predict what treatments should be effective. However, it should be bear in mind that standardization does not mean restriction. For instance, testing a range of regimens, dose levels, inoculation routes, bacterial strains, bacterial growth conditions, animal species, and animal strains will it be possible to determine the most effective treatment for further investigation in a clinical trial—the only way of reaching a definitive conclusion. Nevertheless, clinical trials in the context of plague raise obvious ethical concerns.

## Figures and Tables

**Table 1:** Susceptibility of *Yersinia pestis* to conventional drugs.

Name of the Drug	MIC (µg/mL) at 28 °C			MIC (µg/mL) at 37 °C			References
	Range	CMI_50_	CMI_90_	Range	CMI_50_	CMI_90_	
Streptomycin	1–16 0.125–16 - 4–8 - -	4 2 - 4 - -	8 4 - 4 - -	1–8 - 0.5–8 - 1–8 1.5–4	2 - 4 - - 3	4 - 4 - 4 3	[54,58,59,64,66,67]
Amikacin	0.125–8 0.125–8	2 2	8 4	0.25–4 -	1 -	2 -	[59,67]
Gentamicin	0.06–2 0.125–4 - 0.25–1 - -	1 0.5 - 0.5 - -	2 2 - 1 - -	0.06–1 - 0.12–2 - 0.06–4 0.19–1	0.5 - 0.25 - - 0.38	1 - 0.5 - 1 0.75	[54,58,59,64,66,67]
Doxycycline	0.06–0.5 0.125–2 - 0.25–1 - 0.25–0.5 -	0.25 0.5 - 0.5 - 0.5 -	0.5 1 - 1 - 0.5 -	0.06–2 - 0.12–1 - 0.12–4 - 0.125–2	0.5 - 0.5 - - - 1	1 - 1 - 2 - 1.5	[58,59,60,64,66,67]
Tetracycline	1–16 - 0.5–4 -	4 - 2 -	8 - 4 -	0.25–2 0.25–2 - 0.25–4	0.5 1 - -	2 1 - 2	[54,64,66,67]
Trimethoprime/ Sulfamethoxazole	0.12/2–8/>64 0.25/4.7–2/38 - 0.5/2–1/32 - -	0.5/16 1/19 - 0.5/8 - -	2/>64 2/38 - 1/16 - -	0.12/0.5–16/>64 - 0.03/0.6–0.25/4.75 - ≤0.015–0.25 0.012–0.047	0.5/16 - 0.06/1.19 - - 0.023	8/64 - 0.12/2.38 - 0.06 0.032	[54,58,59,64,66,67]
Rifampin	1–64 2–8 -	2 4 -	16 8 -	0.25–4 - 2–32	2 - 8	4 - 16	[54,58,67]
Chloramphenicol	0.5–16 0.125–8 - 0.5–4 -	4 4 - 4 -	8 4 - 8 -	0.25–4 - 0.5–4 - 0.25–4	1 - 2 - 1.5	4 - 4 - 4	[54,58,59,66,67]
Amoxicillin	0.06–1 0.125–0.5	0.25 0.5	0.5 0.5	0.03–1 -	0.25 -	0.5 -	[59,67]
Cefotaxime	0.004–0.015 <0.125–0.125	0.008 <0.125	0.015 <0.125	0.004–0.03 -	0.015 -	0.03 -	[59,67]
Imipenem	0.06–1 0.125–0.5 -	0.25 0.25 -	0.5 0.5 -	0.12–1 - 0.0094- >32	0.25 - 0.5	0.5 - >32	[58,59,67]
Levofloxacin	0.008–0.06 - -	0.03 - -	0.03 - -	0.008–0.12 0.03–0.06 ≤0.06	0.03 0.03 -	0.06 0.03 1	[64,66,67]
Ciprofloxacin	0.004–0.03 <0.125–0.125 - 0.008–0.062 - 0.016–0.031	0.008 <0.125 - 0.031 - 0.031	0.015 <0.125 - 0.062 - 0.031	0.008–0.12 - 0.03–0.12 - ≤0.03–0.5 -	0.015 - 0.03 - - -	0.03 - 0.03 - 0.12 -	[54,59,60,64,66,67]

**Table 2:** Antibiotic treatment of experimental plague.

Animal Model	*Y. pestis* Strain	Route of Challenge and Infective Dose	Antibiotics	Route of Drugs Administration and Duration in Days	Post Challenge Initiation of Antibiotic Regimen	Death Rates in %	Ref.
Mouse	NA	Subcutaneous 75–150 CFU	PEN STR Sulfathiazole Sulfapyridine Sulfadiazine Sulfamerazine Sulfamethazine	Subcutaneous 5 to 10 days	48 h	100 0 10 100 50 30 30	[75]
			PEN STR Sulfathiazole Sulfapyridine Sulfadiazine Sulfamerazine Sulfamethazine		72 h	100 10 30 100 60 30 30	
Mouse	CO92	Subcutaneous 1.9 × 10^4^ to 2.85 × 10^4^	STR CRO	Intraperitoneal (5 days)	24 h 42 h 48 h 54 h 24 h 42 h 48 h 54 h	0 30 75 55 30 100 100 100	[56]
Mouse	GB	Subcutaneous 1.08 × 10^6^	CIP GAT MXF	Oral (7 days)	1 h 6 h 18 h 24 h	0 for all antibiotics 0 for all antibiotics 17 (CIP), 11 (GAT), 45 (MXF) 72 (CIP), 28 (GAT), 67 (MXF)	[61]
Mouse	CO92	Intradermal ~ 100	GEN CIP GEN + CIP GEN CIP GEN + CIP	Intravenous (5 days)	44 h 56 h	15 0 0 17 12 0	[74]
Mouse	CO92	Aerosol 2.3 × 10^6^ ± 1.15 × 10^6^	CIP OFL STR GEN NET CRO CAZ AMP RIF	Intraperitoneal (5 days)	24 h	0 0 0 20 0 0 15 15 10	[56]
			CIP OFL STR GEN NET CRO CAZ AMP RIF		42 h	38 40 41 15 40 98 100 95 80	
Mouse	GB C092	Aerosol 8.4 × 10^5^ ± 4.2 × 10^4^ 1.9 × 10^5^ ± 7.4 × 10^3^	CIP DOX	Subcutaneous (5 days)	24 h 48 h 24 h 48 h	0 10079 (GB) and-86 (CO92) 100	[90]
Mouse	GB	Aerosol 6 × 10^8^	CIP GAT MXF	Oral (7 days)	6 h 18 h 30 h 48 h	0 for all antibiotics 0 for all antibiotics 0 for all antibiotics 94 (CIP), 67 (GAT) 56 (MXF)	[61]
Mouse	CO92	Aerosol 4.6 × 10^5^	DOX LVX GEN	Intraperitoneal (5 days)	24 h	10 0 10–20	[63]
Mouse	CO92	Aerosol 8.5 × 10^3^	LVX	Intraperitoneal (6 days)	24 h 36 h 48 h	0 with dose ≥ 5 mg/kg/day 10 with dose ≥ 5 mg/kg/day 80–90 with dose ≥ 5 mg/kg/day	[111]
Mouse	CO92	Aerosol 1.36 × 10^6^	IPM CAZ CIPIPM CAZ CIP	Intraperitoneal (5 days)	24 h 42 h	10 10 070 90 50	[108]
Mouse	CO92	Aerosol 2 × 10^6^	OMC DOX CIP	Intraperitoneal (7 days)	24 h	10 10 0	[69]
Rat	C092	Aerosol 7 × 10^3^ 1.25 × 10^4^	LVX Cethromycin	Intraperitoneal (6 days) Oral (7 days)	24 h 36 h 42 h 48 h 24 h 36 h 48 h 60 h	0 0 0 56 0 56 56 100	[107]
Non-human primates	CO92	Aerosol ~3.5 × 10^4^	LVX	Intravenous (10 days)	Within 6 h of the appearance of fever ≥ 39 °C more than 1 h	0	[106]
Non-human primates	CO92	Aerosol 3.5 × 10^4^ ± 1.75 × 10^4^	PLZ	Intravenous (10 days)	Within 6 h of the appearance of fever ≥39 °C more than 1 h	8 (dose of 25/mg/kg)	[109]
Non-human primates	CO92	Aerosol ~3.43 × 10^4^	GEN CIP LVX DOX	Intravenous (10 days) Intravenous (10 days) Intravenous (10 days) Intravenous or oral (10 days)	Within 6 h of the appearance of fever ≥39 °C more than 1 h	20 to 40 according dose 0 to 10 according dose 6 25 to 100 according dose and route of administration	[112]
Mouse	GB	Intraperitoneal 7.3 × 10^2^	TVA GRX	Oral (7 days)	24 h 48 h 72 h 24 h 48 h 72 h	0 20 30 10 50 50	[100]
Mouse	GB	Intraperitoneal 2.28 × 10 to 2.28 × 10^6^ Intraperitoneal 2.94 × 10^2^ to 2.94 × 10^5^	CIP DOX	Subcutaneous (5 days)	24 h	0 at 2.28 × 10 – 2.28 × 10^2^ CFU 20 at 2.28 × 10^3^ CFU 60 to 100 at >2.28 × 10^3^ CFU 80 to 100 at ≥2.94 ×10^2^ CFU	[86]
Mouse	6/69	Intravenous 1000 ± 200	CHL STR	Intramuscular 2 days 3 days 4 days Subcutaneous 2 days 3 days 4 days	24 h 24 h	100 100 20 20 0 0	[91]

PEN: penicillin, STR: streptomycin, CRO: ceftriaxone, CIP: ciprofloxacin, GAT: gatifloxacin, MXF: moxifloxacin, GEN: gentamicin, OFL: ofloxacin, NET: netilmicin, CAZ: ceftazidime, AMP: ampicillin, RIP: rifampin, DOX: doxycycline, LVX: levofloxacin, IPM: imipenem, OMC: omadacycline, PLZ: plazomicin GRX: grepafloxacin, CHL: chloramphenicol.

## Data Availability

Not applicable.

## References

[1] Bos K.I., Schuenemann V.J., Golding G.B., Burbano H.A., Waglechner N., Coombes B.K., McPhee J.B., DeWitte S.N., Meyer M., Schmedes S. (2011). A draft genome of *Yersinia pestis* from victims of the Black Death. Nature.

[2] Feldman M., Harbeck M., Keller M., Spyrou M.A., Rott A., Trautmann B., Scholz H.C., Paffgen B., Peters J., McCormick M. (2016). A High-Coverage *Yersinia pestis* Genome from a Sixth-Century Justinianic Plague Victim. Mol. Biol. Evol..

[3] Haensch S., Bianucci R., Signoli M., Rajerison M., Schultz M., Kacki S., Vermunt M., Weston D.A., Hurst D., Achtman M. (2010). Distinct clones of *Yersinia pestis* caused the black death. PLoS Pathog..

[4] Harbeck M., Seifert L., Hansch S., Wagner D.M., Birdsell D., Parise K.L., Wiechmann I., Grupe G., Thomas A., Keim P. (2013). *Yersinia pestis* DNA from skeletal remains from the 6th century AD reveals insights into Justinianic Plague. PLoS Pathog..

[5] Namouchi A., Guellil M., Kersten O., Hansch S., Ottoni C., Schmid B.V., Pacciani E., Quaglia L., Vermunt M., Bauer E.L. (2018). Integrative approach using *Yersinia pestis* genomes to revisit the historical landscape of plague during the Medieval Period. Proc. Natl. Acad. Sci. USA.

[6] Spyrou M.A., Tukhbatova R.I., Feldman M., Drath J., Kacki S., Beltran de Heredia J., Arnold S., Sitdikov A.G., Castex D., Wahl J. (2016). Historical *Y. pestis* Genomes Reveal the European Black Death as the Source of Ancient and Modern Plague Pandemics. Cell Host Microbe.

[7] Wagner D.M., Klunk J., Harbeck M., Devault A., Waglechner N., Sahl J.W., Enk J., Birdsell D.N., Kuch M., Lumibao C. (2014). *Yersinia pestis* and the plague of Justinian 541-543 AD: A genomic analysis. Lancet Infect. Dis..

[8] Bramanti B., Dean K.R., Walloe L., Chr Stenseth N. (2019). The Third Plague Pandemic in Europe. Proc. Biol. Sci..

[9] Bertherat E. (2019). Plague around the world in 2019. World Health Organization. Wkly. Epidemiol. Rec..

[10] Andrianaivoarimanana V., Kreppel K., Elissa N., Duplantier J.M., Carniel E., Rajerison M., Jambou R. (2013). Understanding the persistence of plague foci in Madagascar. PLoS Negl. Trop. Dis..

[11] Bitam I., Ayyadurai S., Kernif T., Chetta M., Boulaghman N., Raoult D., Drancourt M. (2010). New rural focus of plague, Algeria. Emerg. Infect. Dis..

[12] Cabanel N., Leclercq A., Chenal-Francisque V., Annajar B., Rajerison M., Bekkhoucha S., Bertherat E., Carniel E. (2013). Plague outbreak in Libya, 2009, unrelated to plague in Algeria. Emerg. Infect. Dis..

[13] Eurasianet.org. http://www.eurasianet.org/node/67429.

[14] Sherman I.W. (2007). Twelve Diseases that Changed Our World.

[15] Galimand M., Guiyoule A., Gerbaud G., Rasoamanana B., Chanteau S., Carniel E., Courvalin P. (1997). Multidrug resistance in *Yersinia pestis* mediated by a transferable plasmid. N. Engl. J. Med..

[16] Kiefer D., Dalantai G., Damdindorj T., Riehm J.M., Tomaso H., Zoller L., Dashdavaa O., Pfister K., Scholz H.C. (2012). Phenotypical characterization of Mongolian *Yersinia pestis* strains. Vector Borne Zoonotic Dis..

[17] Butler T. (1983). Plague and Other Yersinia Infections.

[18] Flexner S. (1901). The pathology of bubonic plague. Am. J. Med. Sci..

[19] Crowell B.C. (1915). Pathologic anatomy of bubonic plague. Philipp. J. Sci..

[20] Hull H.F., Montes J.M., Mann J.M. (1987). Septicemic plague in New Mexico. J. Infect. Dis..

[21] Sebbane F., Jarrett C.O., Gardner D., Long D., Hinnebusch B.J. (2006). Role of the *Yersinia pestis* plasminogen activator in the incidence of distinct septicemic and bubonic forms of flea-borne plague. Proc. Natl. Acad. Sci. USA.

[22] Kugeler K.J., Mead P.S., Campbell S.B., Nelson C.A. (2020). Antimicrobial Treatment Patterns and Illness Outcome Among United States Patients With Plague, 1942–2018. Clin. Infect. Dis..

[23] Godfred-Cato S., Cooley K.M., Fleck-Derderian S., Becksted H.A., Russell Z., Meaney-Delman D., Mead P.S., Nelson C.A. (2020). Treatment of Human Plague: A Systematic Review of Published Aggregate Data on Antimicrobial Efficacy, 1939–2019. Clin. Infect. Dis..

[24] Nikiforov V.V., Gao H., Zhou L., Anisimov A. (2016). Plague: Clinics, Diagnosis and Treatment. Adv. Exp. Med. Biol..

[25] Fonquernie J. (1931). Observations sur un cas de Pestis minor. Bull. Soc. Pathol. Exot..

[26] Pollitzer R. (1954). Plague.

[27] Leslie T., Whitehouse C.A., Yingst S., Baldwin C., Kakar F., Mofleh J., Hami A.S., Mustafa L., Omar F., Ayazi E. (2011). Outbreak of gastroenteritis caused by *Yersinia pestis* in Afghanistan. Epidemiol. Infect..

[28] Butler T., Fu Y.S., Furman L., Almeida C., Almeida A. (1982). Experimental *Yersinia pestis* infection in rodents after intragastric inoculation and ingestion of bacteria. Infect. Immun..

[29] Becksted T.M., Poland J.D., Quan T.J., White M.E., Mann J.M., Barnes A.M. (1987). Plague meningitis--a retrospective analysis of cases reported in the United States, 1970–1979. West. J. Med..

[30] Williams J.E., Harrison D.N., Quan T.J., Mullins J.L., Barnes A.M., Cavanaugh D.C. (1978). Atypical plague bacilli isolated from rodents, fleas, and man. Am. J. Public Health.

[31] Sebbane F., Jarrett C., Gardner D., Long D., Hinnebusch B.J. (2009). The *Yersinia pestis caf1M1A1* fimbrial capsule operon promotes transmission by flea bite in a mouse model of bubonic plague. Infect. Immun..

[32] Sebbane F., Jarrett C., Gardner D., Long D., Hinnebusch B.J. (2010). Role of the *Yersinia pestis* yersiniabactin iron acquisition system in the incidence of flea-borne plague. PLoS ONE.

[33] Simond P. (1898). La propagation de la peste. Ann. Inst. Pasteur.

[34] Sebbane F., Gardner D., Long D., Gowen B.B., Hinnebusch B.J. (2005). Kinetics of disease progression and host response in a rat model of bubonic plague. Am. J. Pathol..

[35] Bosio C.F., Jarrett C.O., Scott D.P., Fintzi J., Hinnebusch B.J. (2020). Comparison of the transmission efficiency and plague progression dynamics associated with two mechanisms by which fleas transmit *Yersinia pestis*. PLoS Pathog..

[36] Strong R.P., Crowell B.C., Teague O. (1912). Studies on pneumonic plague and plague immunization. Philipp. J. Sci..

[37] Lathem W.W., Crosby S.D., Miller V.L., Goldman W.E. (2005). Progression of primary pneumonic plague: A mouse model of infection, pathology, and bacterial transcriptional activity. Proc. Natl. Acad. Sci. USA.

[38] Lathem W.W., Price P.A., Miller V.L., Goldman W.E. (2007). A plasminogen-activating protease specifically controls the development of primary pneumonic plague. Science.

[39] Caulfield A.J., Walker M.E., Gielda L.M., Lathem W.W. (2014). The Pla protease of *Yersinia pestis* degrades fas ligand to manipulate host cell death and inflammation. Cell Host Microbe.

[40] Zimbler D.L., Schroeder J.A., Eddy J.L., Lathem W.W. (2015). Early emergence of *Yersinia pestis* as a severe respiratory pathogen. Nat. Commun..

[41] Anisimov A.P., Amoako K.K. (2006). Treatment of plague: Promising alternatives to antibiotics. J. Med. Microbiol..

[42] Harrison F., Roberts A.E., Gabrilska R., Rumbaugh K.P., Lee C., Diggle S.P. (2015). A 1,000-Year-Old Antimicrobial Remedy with Antistaphylococcal Activity. mBio.

[43] Fleming A. (1929). On the antibacterial action of cultures of a penicillium, with special reference to their use in the isolation of B. influenzae. Br. J. Exp. Pathol..

[44] Hutchings M.I., Truman A.W., Wilkinson B. (2019). Antibiotics: Past, present and future. Curr. Opin. Microbiol..

[45] Kugeler K.J., Staples J.E., Hinckley A.F., Gage K.L., Mead P.S. (2015). Epidemiology of human plague in the United States, 1900–2012. Emerg Infect Dis.

[46] Butler T. (2014). Plague history: Yersin’s discovery of the causative bacterium in 1894 enabled, in the subsequent century, scientific progress in understanding the disease and the development of treatments and vaccines. Clin. Microbiol. Infect..

[47] Prentice M.B., Rahalison L. (2007). Plague. Lancet.

[48] Salam A.P., Rojek A., Cai E., Raberahona M., Horby P. (2020). Deaths Associated with Pneumonic Plague, 1946–2017. Emerg. Infect. Dis..

[49] Randremanana R., Andrianaivoarimanana V., Nikolay B., Ramasindrazana B., Paireau J., Ten Bosch Q.A., Rakotondramanga J.M., Rahajandraibe S., Rahelinirina S., Rakotomanana F. (2019). Epidemiological characteristics of an urban plague epidemic in Madagascar, August-November, 2017: An outbreak report. Lancet Infect. Dis..

[50] Rasoamanana B., Coulanges P., Michel P., Rasolofonirina N. (1989). Sensitivity of *Yersinia pestis* to antibiotics: 277 strains isolated in Madagascar between 1926 and 1989. Arch. Inst. Pasteur Madag..

[51] Barrow E.W., Clinkenbeard P.A., Duncan-Decocq R.A., Perteet R.F., Hill K.D., Bourne P.C., Valderas M.W., Bourne C.R., Clarkson N.L., Clinkenbeard K.D. (2012). High-throughput screening of a diversity collection using biodefense category A and B priority pathogens. J. Biomol. Screen.

[52] Bonacorsi S.P., Scavizzi M.R., Guiyoule A., Amouroux J.H., Carniel E. (1994). Assessment of a fluoroquinolone, three beta-lactams, two aminoglycosides, and a cycline in treatment of murine *Yersinia pestis* infection. Antimicrob. Agents Chemother..

[53] Goto S., Tsuji A., Murai T., Nishida M., Tsukano H., Watanabe H. (1995). In vitro activity of twenty-three antimicrobials against *Yersinia pestis* strains. J. Infect. Chemother..

[54] Smith M.D., Vinh D.X., Nguyen T.T., Wain J., Thung D., White N.J. (1995). In vitro antimicrobial susceptibilities of strains of *Yersinia pestis*. Antimicrob. Agents Chemother..

[55] Frean J.A., Arntzen L., Capper T., Bryskier A., Klugman K.P. (1996). In vitro activities of 14 antibiotics against 100 human isolates of *Yersinia pestis* from a southern African plague focus. Antimicrob. Agents Chemother..

[56] Byrne W.R., Welkos S.L., Pitt M.L., Davis K.J., Brueckner R.P., Ezzell J.W., Nelson G.O., Vaccaro J.R., Battersby L.C., Friedlander A.M. (1998). Antibiotic treatment of experimental pneumonic plague in mice. Antimicrob. Agents Chemother..

[57] Paetzel M., Dalbey R.E., Strynadka N.C. (2000). The structure and mechanism of bacterial type I signal peptidases. A novel antibiotic target. Pharmacol. Ther..

[58] Wong J.D., Barash J.R., Sandfort R.F., Janda J.M. (2000). Susceptibilities of *Yersinia pestis* strains to 12 antimicrobial agents. Antimicrob. Agents Chemother..

[59] Hernandez E., Girardet M., Ramisse F., Vidal D., Cavallo J.D. (2003). Antibiotic susceptibilities of 94 isolates of *Yersinia pestis* to 24 antimicrobial agents. J. Antimicrob. Chemother..

[60] Frean J., Klugman K.P., Arntzen L., Bukofzer S. (2003). Susceptibility of *Yersinia pestis* to novel and conventional antimicrobial agents. J. Antimicrob. Chemother..

[61] Steward J., Lever M.S., Russell P., Beedham R.J., Stagg A.J., Taylor R.R., Brooks T.J. (2004). Efficacy of the latest fluoroquinolones against experimental *Yersinia pestis*. Int. J. Antimicrob. Agents.

[62] Louie A., Deziel M.R., Liu W., Drusano G.L. (2007). Impact of resistance selection and mutant growth fitness on the relative efficacies of streptomycin and levofloxacin for plague therapy. Antimicrob. Agents Chemother..

[63] Heine H.S., Louie A., Sorgel F., Bassett J., Miller L., Sullivan L.J., Kinzig-Schippers M., Drusano G.L. (2007). Comparison of 2 antibiotics that inhibit protein synthesis for the treatment of infection with *Yersinia pestis* delivered by aerosol in a mouse model of pneumonic plague. J. Infect. Dis..

[64] Lonsway D.R., Urich S.K., Heine H.S., McAllister S.K., Banerjee S.N., Schriefer M.E., Patel J.B. (2011). Comparison of Etest method with reference broth microdilution method for antimicrobial susceptibility testing of *Yersinia pestis*. J. Clin. Microbiol..

[65] Louie A., Heine H.S., VanScoy B., Eichas A., Files K., Fikes S., Brown D.L., Liu W., Kinzig-Schippers M., Sorgel F. (2011). Use of an in vitro pharmacodynamic model to derive a moxifloxacin regimen that optimizes kill of *Yersinia pestis* and prevents emergence of resistance. Antimicrob. Agents. Chemother..

[66] Urich S.K., Chalcraft L., Schriefer M.E., Yockey B.M., Petersen J.M. (2012). Lack of antimicrobial resistance in *Yersinia pestis* isolates from 17 countries in the Americas, Africa, and Asia. Antimicrob. Agents Chemother..

[67] Heine H.S., Hershfield J., Marchand C., Miller L., Halasohoris S., Purcell B.K., Worsham P.L. (2015). In vitro antibiotic susceptibilities of *Yersinia pestis* determined by broth microdilution following CLSI methods. Antimicrob. Agents Chemother..

[68] Steed D.B., Liu J., Wasbrough E., Miller L., Halasohoris S., Miller J., Somerville B., Hershfield J.R., Romesberg F.E. (2015). Origins of *Yersinia pestis* sensitivity to the arylomycin antibiotics and the inhibition of type I signal peptidase. Antimicrob. Agents Chemother..

[69] Steenbergen J., Tanaka S.K., Miller L.L., Halasohoris S.A., Hershfield J.R. (2017). In Vitro and In Vivo Activity of Omadacycline against Two Biothreat Pathogens, *Bacillus anthracis* and *Yersinia pestis*. Antimicrob. Agents Chemother..

[70] Smith P.A., Koehler M.F.T., Girgis H.S., Yan D., Chen Y., Chen Y., Crawford J.J., Durk M.R., Higuchi R.I., Kang J. (2018). Optimized arylomycins are a new class of Gram-negative antibiotics. Nature.

[71] Scarff J.M., Waidyarachchi S.L., Meyer C.J., Lane D.J., Chai W., Lemmon M.M., Liu J., Butler M.M., Bowlin T.L., Lee R.E. (2019). Aminomethyl spectinomycins: A novel antibacterial chemotype for biothreat pathogens. J. Antibiot..

[72] Rebeil R., Ernst R.K., Gowen B.B., Miller S.I., Hinnebusch B.J. (2004). Variation in lipid A structure in the pathogenic *Yersiniae*. Mol. Microbiol..

[73] Louie A., Vanscoy B., Liu W., Kulawy R., Brown D., Heine H.S., Drusano G.L. (2011). Comparative efficacies of candidate antibiotics against *Yersinia pestis* in an in vitro pharmacodynamic model. Antimicrob. Agents Chemother..

[74] Lemaitre N., Ricard I., Pradel E., Foligne B., Courcol R., Simonet M., Sebbane F. (2012). Efficacy of ciprofloxacin-gentamicin combination therapy in murine bubonic plague. PLoS ONE.

[75] Sokhey S.S., Wagle P.M., Habbu M.K. (1953). Treatment of bubonic plague with sulfonamides and antibiotics. Bull. World Health Organ..

[76] Kalininskii V.B., Vasil’ev N.T., Iudin S.M. (1989). Effectiveness of quinolones against *Y. pestis*. Antibiot. Khimioter..

[77] Shcherbaniuk A.I., Makarovskaia L.N., Bugaeva O.K., Kasatkina I.V. (1992). Antibiotics of the aminoglycoside group (gentamicin, sisomicin and amikacin) in the prevention and treatment of experimental plague. Antibiot. Khimioter..

[78] Samokhodkina E.D., Ryzhko I.V., Shcherbaniuk A.I., Kasatkina I.V., Tsuraeva R.I., Zhigalova T.A. (1992). Doxycycline in the prevention of experimental plague induced by plague microbe variants. Antibiot. Khimioter..

[79] Kasatkina I.V., Sherbaniuk A.I., Makarovskaia L.N., Padeiskaia E.N. (1993). Effectiveness of the new quinolones, ciprofloxacin and pefloxacin in experimental plague. Antibiot. Khimioter..

[80] Makarovskaia L.N., Shcherbaniuk A.I., Bugaeva O.K. (1993). Efficacy of rifampicin in experimental plague infection. Antibiot. Khimioter..

[81] Ryzhko I.V., Samokhodkina E.D., Zhigalova T.A. (1993). Ceftriaxone in prevention and treatment of experimental plague infection. Antibiot. Khimioter..

[82] Pasiukov V.V., Ryzhko I.V., Tsuraeva R.I., Samokhodkina E.D., Roshchina N.V. (1994). Cefoperazone in the prevention and treatment of experimental plague caused by typical and fraction-free pathogen strains in white mice. Antibiot. Khimioter..

[83] Samokhodkina E.D., Ryzhko I.V., Tsuraeva R.I., Pasiukov V.V. (1994). Beta-lactam antibiotics (ampicillin, cefotaxime) in prevention of experimental plague in albino mice, caused by non-fractioned strains of the pathogen. Antibiot. Khimioter..

[84] Romanov V.E., Vasil’ev N.T., Shabalin B.A., Kozhemiako A.V., Zhivov I.V. (1995). Methods for rapid evaluation of the effectiveness of drugs showing promise for urgent prevention and treatment of plague. Antibiot. Khimioter..

[85] Makarovskaia L.N., Ryzhkova V.V., Zurabian V.A., Bugaeva O.K., Pasiukov V.V., Fomina I.P., Navashin S.M. (1995). Comparative effectiveness of rifampicin for parenteral administration and internal dose in the treatment of experimental plague in albino mice. Antibiot. Khimioter..

[86] Russell P., Eley S.M., Bell D.L., Manchee R.J., Titball R.W. (1996). Doxycycline or ciprofloxacin prophylaxis and therapy against experimental *Yersinia pestis* infection in mice. J. Antimicrob. Chemother..

[87] Ryzhko I.V., Tsuraeva R.I., Pasiukov V.V., Samokhodkina E.D., Shcherbaniuk A.I. (1996). Third generation cephalosporins (cefoperazone, cefotaxime, ceftazidime and ceftriaxone) in the prevention and treatment of experimental plague in albino mice. Antibiot. Khimioter..

[88] Ryzhko I.V., Shcherbaniuk A.I., Tsuraeva R.I., Samokhodkina E.D., Kasatkina I.V., Tsetskhladze N.S., Pasiukov V.V., Roshchina N.V. (1997). A comparative study of fluoroquinolones and 3rd-generation cephalosporins in the prevention and treatment of experimental plague caused by *Yersinia pestis* strains typical and serologically atypical with respect to F1. Antibiot. Khimioter..

[89] Goto S., Tsuji A., Murai T., Nishida M., Tsukano H., Watanabe H. (1998). Therapeutic effect of antimicrobial drugs against experimental infections due to *Yersinia pestis* in mice. J. Infect. Chemother..

[90] Russell P., Eley S.M., Green M., Stagg A.J., Taylor R.R., Nelson M., Beedham R.J., Bell D.L., Rogers D., Whittington D. (1998). Efficacy of doxycycline and ciprofloxacin against experimental *Yersinia pestis* infection. J. Antimicrob. Chemother..

[91] Rahalison L., Guiyoule A., Bonacorsi S.P., Slacanin I., Chanteau S., Carniel E. (2000). Failure of oily chloramphenicol depot injection to treat plague in a murine model. J. Antimicrob. Chemother..

[92] Romanov V.E., Vasil’ev N.T., Shabalin B.A., Mironin A.V. (2001). Effect of antibacterial therapy on the epidemic threat of experimental pneumonic plague in monkeys. Antibiot. Khimioter..

[93] Samokhodkina E.D., Shcherbaniuk A.I., Ryzhko I.V., Tsuraeva R.I., Skalyga E. (2002). Ofloxacin efficacy in the prophylaxis and treatment of experimental plague due to antigen complete and defective strains of the pathogen. Antibiot. Khimioter..

[94] Romanov V.E., Evstigneev V.I., Vasil’ev N.T., Shabalin B.A., Paramonov V.E. (2001). Evaluation of the effectiveness of antibacterial substances in treating an experimental form of bubonic plague in monkeys. Antibiot. Khimioter..

[95] Ryzhko I.V., Tsuraeva R.I., Moldavan I.A., Shcherbaniuk A.I. (2004). Efficacy of plague prophylaxis with streptomycin, tetracycline, and rifampicin in simultaneous immunization of white mice by resistant EV NRIEG strain. Antibiot. Khimioter..

[96] Smorodinova Iu V., Shcherbaniuk A.I., Ryzhko I.V., Moldavan I.A. (2005). Isepamycin in prophylaxis and treatment of experimental plague due to FI+ and FI- variants of plague microbe. Antibiot. Khimioter..

[97] Shcherbaniuk A.I., Liukshina E., Ryzhko I.V., Smorodinova Iu V. (2006). Synergistic action of some antibacterial agents in studies on albino mice with experimental plague caused by Fr- phenotype strain of the plague microbe. Antibiot. Khimioter..

[98] Ryzhko I.V., Tsuraeva R.I., Anisimov B.I., Trishina A.V. (2009). Efficacy of levofloxacin, lomefloxacin and moxifloxacin vs. other fluoroquinolones in experimental plague due to FI+ and FI- strains of *Yersinia pestis* in Albino mice. Antibiot. Khimioter..

[99] Tleyjeh I.M., Kashour T., Hakim F.A., Zimmerman V.A., Erwin P.J., Sutton A.J., Ibrahim T. (2009). Statins for the prevention and treatment of infections: A systematic review and meta-analysis. Arch. Intern. Med..

[100] Kenny D.J., Sefton A.M., Steward J., Brooks T.J., Simpson A.J., Atkins H.S. (2009). Efficacy of the quinolones trovafloxacin and grepafloxacin for therapy of experimental tularaemia and plague. Int. J. Antimicrob. Agents.

[101] Ayyadurai S., Lepidi H., Nappez C., Raoult D., Drancourt M. (2010). Lovastatin protects against experimental plague in mice. PLoS ONE.

[102] Ryzhko I.V., Shcherbaniuk A.I., Moldavan I.A., Tsuraeva R.I., Anisimov B.I., Trishina A.V. (2007). [Efficacy of cefixime and cefepime vs. other cephalosporins in experimental plague of albino mice due to variants FI+ and FI- of the plague microbe]. Antibiot. Khimioter..

[103] Ryzhko I.V., Moldavan I.A., Tsuraeva R.I., Shcherbaniuk A.I. (2006). Prophylactic use of ceftriaxone in combination with F I antigen immunization in studies on uninbred albino mice infected by *Yersinia pestis*. Antiplague immunity development. Antibiot. Khimioter..

[104] Panchal R.G., Ulrich R.L., Lane D., Butler M.M., Houseweart C., Opperman T., Williams J.D., Peet N.P., Moir D.T., Nguyen T. (2009). Novel broad-spectrum bis-(imidazolinylindole) derivatives with potent antibacterial activities against antibiotic-resistant strains. Antimicrob. Agents Chemother..

[105] Ryzhko I.V., Trishina A.V., Verkina L.M. (2010). Lack of levofloxacin and moxyfloxacin efficacy in experimental plague of albino mice infected with nalidixic acid resistant pathogen (Nal(r)). Antibiot. Khimioter..

[106] Layton R.C., Mega W., McDonald J.D., Brasel T.L., Barr E.B., Gigliotti A.P., Koster F. (2011). Levofloxacin cures experimental pneumonic plague in African green monkeys. PLoS Negl. Trop. Dis..

[107] Rosenzweig J.A., Brackman S.M., Kirtley M.L., Sha J., Erova T.E., Yeager L.A., Peterson J.W., Xu Z.Q., Chopra A.K. (2011). Cethromycin-mediated protection against the plague pathogen *Yersinia pestis* in a rat model of infection and comparison with levofloxacin. Antimicrob. Agents Chemother..

[108] Heine H.S., Louie A., Adamovicz J.J., Amemiya K., Fast R.L., Miller L., Opal S.M., Palardy J., Parejo N.A., Sorgel F. (2014). Evaluation of imipenem for prophylaxis and therapy of *Yersinia pestis* delivered by aerosol in a mouse model of pneumonic plague. Antimicrob. Agents Chemother..

[109] Mega W.M., Doyle-Eisele M., Cass R.T., Kostrub C.F., Sherwood R.L., Metz M.A., Cirz R.T. (2016). Plazomicin is effective in a non-human primate pneumonic plague model. Bioorg. Med. Chem..

[110] Gur D., Glinert I., Aftalion M., Vagima Y., Levy Y., Rotem S., Zauberman A., Tidhar A., Tal A., Maoz S. (2018). Inhalational Gentamicin Treatment Is Effective Against Pneumonic Plague in a Mouse Model. Front. Microbiol..

[111] Peterson J.W., Moen S.T., Healy D., Pawlik J.E., Taormina J., Hardcastle J., Thomas J.M., Lawrence W.S., Ponce C., Chatuev B.M. (2010). Protection Afforded by Fluoroquinolones in Animal Models of Respiratory Infections with *Bacillus anthracis*, *Yersinia pestis*, and *Francisella tularensis*. Open Microbiol. J..

[112] Hewitt J.A., Lanning L.L., Campbell J.L. (2020). The African Green Monkey Model of Pneumonic Plague and US Food and Drug Administration Approval of Antimicrobials Under the Animal Rule. Clin Infect Dis.

[113] Anisimov A.P., Dyatlov I.A. (1997). A novel mechanism of antibiotic resistance in plague. J. Med. Microbiol..

[114] Meka-Mechenko T.V. (2003). F1-negative natural *Y. pestis* strains. Adv. Exp. Med. Biol..

[115] Winter C., Cherry W., Moody M. (1960). An unusual strain of *Pasteurella pestis* isolated from a fatal human case of plague. Bull. World Health Organ..

[116] Anisimov A.P., Lindler L.E., Pier G.B. (2004). Intraspecific diversity of *Yersinia pestis*. Clin. Microbiol. Rev..

[117] Friedlander A.M., Welkos S.L., Worsham P.L., Andrews G.P., Heath D.G., Anderson G.W., Pitt M.L., Estep J., Davis K. (1995). Relationship between virulence and immunity as revealed in recent studies of the F1 capsule of *Yersinia pestis*. Clin. Infect. Dis..

[118] Cornelius C.A., Quenee L.E., Elli D., Ciletti N.A., Schneewind O. (2009). *Yersinia pestis* IS1541 transposition provides for escape from plague immunity. Infect. Immun..

[119] Andes D., Craig W.A. (2002). Animal model pharmacokinetics and pharmacodynamics: A critical review. Int. J. Antimicrob. Agents.

[120] Cipolla D., Chan H.K. (2013). Inhaled antibiotics to treat lung infection. Pharm. Pat. Anal..

[121] Mwengee W., Butler T., Mgema S., Mhina G., Almasi Y., Bradley C., Formanik J.B., Rochester C.G. (2006). Treatment of plague with gentamicin or doxycycline in a randomized clinical trial in Tanzania. Clin. Infect. Dis..

[122] Randremanana R.V., Raberahona M., Randria M.J.D., Rajerison M., Andrianaivoarimanana V., Legrand A., Rasoanaivo T.F., Randriamparany R., Mayouya-Gamana T., Mangahasimbola R. (2020). An open-label, randomized, non-inferiority trial of the efficacy and safety of ciprofloxacin versus streptomycin + ciprofloxacin in the treatment of bubonic plague (IMASOY): Study protocol for a randomized control trial. Trials.

[123] Boulanger L.L., Ettestad P., Fogarty J.D., Dennis D.T., Romig D., Mertz G. (2004). Gentamicin and tetracyclines for the treatment of human plague: Review of 75 cases in new Mexico, 1985–1999. Clin. Infect. Dis..

[124] Apangu T., Griffith K., Abaru J., Candini G., Apio H., Okoth F., Okello R., Kaggwa J., Acayo S., Ezama G. (2017). Successful Treatment of Human Plague with Oral Ciprofloxacin. Emerg. Infect. Dis..

[125] Reynolds A.V., Hamilton-Miller J.M., Brumfitt W. (1976). Diminished effect of gentamicin under anaerobic or hypercapnic conditions. Lancet.

[126] Simon V.K., Mosinger E.U., Malerczy V. (1973). Pharmacokinetic studies of tobramycin and gentamicin. Antimicrob. Agents Chemother..

[127] Drusano G., Labro M.T., Cars O., Mendes P., Shah P., Sorgel F., Weber W. (1998). Pharmacokinetics and pharmacodynamics of fluoroquinolones. Clin. Microbiol. Infect..

[128] Butler T., Bell W.R., Nguyen Ngoc L., Nguyen Dinh T., Arnold K. (1974). *Yersinia pestis* infection in Vietnam. I. Clinical and hematologic aspects. J. Infect. Dis..

[129] P.a.f.o.b. Emergencies. https://www.fda.gov/drugs/bioterrorism-and-drug-preparedness/products-approved-other-bioterrorism-emergencies.

[130] Bossi P., Tegnell A., Baka A., van Loock F., Werner A., Hendriks J., Maidhof H., Gouvras G. (2004). Bichat guidelines for the clinical management of plague and bioterrorism-related plague. Euro Surveill..

[131] Cabanel N., Bouchier C., Rajerison M., Carniel E. (2018). Plasmid-mediated doxycycline resistance in a *Yersinia pestis* strain isolated from a rat. Int. J. Antimicrob. Agents.

[132] Guiyoule A., Gerbaud G., Buchrieser C., Galimand M., Rahalison L., Chanteau S., Courvalin P., Carniel E. (2001). Transferable plasmid-mediated resistance to streptomycin in a clinical isolate of *Yersinia pestis*. Emerg. Infect. Dis..

[133] Marshall J.D., Joy R.J., Ai N.V., Quy D.V., Stockard J.L., Gibson F.L. (1967). Plague in Vietnam 1965–1966. Am. J. Epidemiol..

[134] Hunter D.R., Dangerfield H.G. (1967). Plague in Vietnam. USARV Med. Bull..

[135] Walter Reed Army Institute Research, U.M.R.T (1966). Vietman and Institut Pasteur of Vietnam. Annu. Prog. Rep..

[136] Lyamuya E.F., Nyanda P., Mohammedali H., Mhalu F.S. (1992). Laboratory studies on *Yersinia pestis* during the 1991 outbreak of plague in Lushoto, Tanzania. J. Trop. Med. Hyg..

[137] Welch T.J., Fricke W.F., McDermott P.F., White D.G., Rosso M.L., Rasko D.A., Mammel M.K., Eppinger M., Rosovitz M.J., Wagner D. (2007). Multiple antimicrobial resistance in plague: An emerging public health risk. PLoS ONE.

[138] Hinnebusch B.J., Rosso M.L., Schwan T.G., Carniel E. (2002). High-frequency conjugative transfer of antibiotic resistance genes to *Yersinia pestis* in the flea midgut. Mol. Microbiol..

[139] Clatworthy A.E., Pierson E., Hung D.T. (2007). Targeting virulence: A new paradigm for antimicrobial therapy. Nat. Chem. Biol..

[140] Dickey S.W., Cheung G.Y.C., Otto M. (2017). Different drugs for bad bugs: Antivirulence strategies in the age of antibiotic resistance. Nat. Rev. Drug Discov..

[141] Ogawara H. (2020). Possible drugs for the treatment of bacterial infections in the future: Anti-virulence drugs. J. Antibiot..

[142] Calvert M.B., Jumde V.R., Titz A. (2018). Pathoblockers or antivirulence drugs as a new option for the treatment of bacterial infections. Beilstein J. Org. Chem..

[143] Onishi H.R., Pelak B.A., Gerckens L.S., Silver L.L., Kahan F.M., Chen M.H., Patchett A.A., Galloway S.M., Hyland S.A., Anderson M.S. (1996). Antibacterial agents that inhibit lipid A biosynthesis. Science.

[144] Tomaras A.P., McPherson C.J., Kuhn M., Carifa A., Mullins L., George D., Desbonnet C., Eidem T.M., Montgomery J.I., Brown M.F. (2014). LpxC inhibitors as new antibacterial agents and tools for studying regulation of lipid A biosynthesis in Gram-negative pathogens. mBio.

[145] Brown M.F., Reilly U., Abramite J.A., Arcari J.T., Oliver R., Barham R.A., Che Y., Chen J.M., Collantes E.M., Chung S.W. (2012). Potent inhibitors of LpxC for the treatment of Gram-negative infections. J. Med. Chem..

[146] Barb A.W., Jiang L., Raetz C.R., Zhou P. (2007). Structure of the deacetylase LpxC bound to the antibiotic CHIR-090: Time-dependent inhibition and specificity in ligand binding. Proc. Natl. Acad. Sci. USA.

[147] Coggins B.E., Li X., McClerren A.L., Hindsgaul O., Raetz C.R., Zhou P. (2003). Structure of the LpxC deacetylase with a bound substrate-analog inhibitor. Nat. Struct. Biol..

[148] Lee C.J., Liang X., Wu Q., Najeeb J., Zhao J., Gopalaswamy R., Titecat M., Sebbane F., Lemaitre N., Toone E.J. (2016). Drug design from the cryptic inhibitor envelope. Nat. Commun..

[149] Lemaitre N., Liang X., Najeeb J., Lee C.J., Titecat M., Leteurtre E., Simonet M., Toone E.J., Zhou P., Sebbane F. (2017). Curative Treatment of Severe Gram-Negative Bacterial Infections by a New Class of Antibiotics Targeting LpxC. mBio.

[150] Titecat M., Liang X., Lee C.J., Charlet A., Hocquet D., Lambert T., Pages J.M., Courcol R., Sebbane F., Toone E.J. (2016). High susceptibility of MDR and XDR Gram-negative pathogens to biphenyl-diacetylene-based difluoromethyl-allo-threonyl-hydroxamate LpxC inhibitors. J. Antimicrob. Chemother..

[151] Kundu R. (2020). Cationic Amphiphilic Peptides: Synthetic Antimicrobial Agents Inspired by Nature. ChemMedChem.

[152] Abdelbaqi S., Deslouches B., Steckbeck J., Montelaro R., Reed D.S. (2016). Novel engineered cationic antimicrobial peptides display broad-spectrum activity against Francisella tularensis, *Yersinia pestis* and *Burkholderia pseudomallei*. J. Med. Microbiol..

[153] Cote C.K., Blanco I.I., Hunter M., Shoe J.L., Klimko C.P., Panchal R.G., Welkos S.L. (2020). Combinations of early generation antibiotics and antimicrobial peptides are effective against a broad spectrum of bacterial biothreat agents. Microb. Pathog..

[154] Lyons B.J.E., Strynadka N.C.J. (2019). On the road to structure-based development of anti-virulence therapeutics targeting the type III secretion system injectisome. MedChemComm.

[155] Morgan J.M., Lam H.N., Delgado J., Luu J., Mohammadi S., Isberg R.R., Wang H., Auerbuch V. (2018). An Experimental Pipeline for Initial Characterization of Bacterial Type III Secretion System Inhibitor Mode of Action Using Enteropathogenic *Yersinia*. Front. Cell Infect. Microbiol..

[156] Dey S., Chakravarty A., Guha Biswas P., De Guzman R.N. (2019). The type III secretion system needle, tip, and translocon. Protein Sci..

[157] Hotinger J.A., May A.E. (2020). Antibodies Inhibiting the Type III Secretion System of Gram-Negative Pathogenic Bacteria. Antibodies.

[158] Viboud G.I., Bliska J.B. (2005). *Yersinia* outer proteins: Role in modulation of host cell signaling responses and pathogenesis. Annu. Rev. Microbiol..

[159] Lemaitre N., Sebbane F., Long D., Hinnebusch B.J. (2006). *Yersinia pestis* YopJ suppresses tumor necrosis factor alpha induction and contributes to apoptosis of immune cells in the lymph node but is not required for virulence in a rat model of bubonic plague. Infect. Immun..

[160] Pan N., Lee C., Goguen J. (2007). High throughput screening for small-molecule inhibitors of type III secretion in *Yersinia pestis*. Adv. Exp. Med. Biol..

[161] Duncan M.C., Linington R.G., Auerbuch V. (2012). Chemical inhibitors of the type three secretion system: Disarming bacterial pathogens. Antimicrob. Agents Chemother..

[162] Kauppi A.M., Nordfelth R., Uvell H., Wolf-Watz H., Elofsson M. (2003). Targeting bacterial virulence: Inhibitors of type III secretion in *Yersinia*. Chem. Biol..

[163] Pan N.J., Brady M.J., Leong J.M., Goguen J.D. (2009). Targeting type III secretion in *Yersinia pestis*. Antimicrob. Agents Chemother..

[164] Nordfelth R., Kauppi A.M., Norberg H.A., Wolf-Watz H., Elofsson M. (2005). Small-molecule inhibitors specifically targeting type III secretion. Infect. Immun..

[165] Jessen D.L., Bradley D.S., Nilles M.L. (2014). A type III secretion system inhibitor targets YopD while revealing differential regulation of secretion in calcium-blind mutants of *Yersinia pestis*. Antimicrob. Agents Chemother..

[166] Marsden A.E., King J.M., Spies M.A., Kim O.K., Yahr T.L. (2016). Inhibition of *Pseudomonas aeruginosa* ExsA DNA-Binding Activity by N-Hydroxybenzimidazoles. Antimicrob. Agents Chemother..

[167] Garrity-Ryan L.K., Kim O.K., Balada-Llasat J.M., Bartlett V.J., Verma A.K., Fisher M.L., Castillo C., Songsungthong W., Tanaka S.K., Levy S.B. (2010). Small molecule inhibitors of LcrF, a Yersinia pseudotuberculosis transcription factor, attenuate virulence and limit infection in a murine pneumonia model. Infect. Immun..

[168] Kim O.K., Garrity-Ryan L.K., Bartlett V.J., Grier M.C., Verma A.K., Medjanis G., Donatelli J.E., Macone A.B., Tanaka S.K., Levy S.B. (2009). N-hydroxybenzimidazole inhibitors of the transcription factor LcrF in *Yersinia*: Novel antivirulence agents. J. Med. Chem..

[169] Huang Z., He Y., Zhang X., Gunawan A., Wu L., Zhang Z.Y., Wong C.F. (2010). Derivatives of salicylic acid as inhibitors of YopH in *Yersinia pestis*. Chem. Biol. Drug Des..

[170] Liang F., Huang Z., Lee S.Y., Liang J., Ivanov M.I., Alonso A., Bliska J.B., Lawrence D.S., Mustelin T., Zhang Z.Y. (2003). Aurintricarboxylic acid blocks in vitro and in vivo activity of YopH, an essential virulent factor of *Yersinia pestis*, the agent of plague. J. Biol. Chem..

[171] Phan J., Lee K., Cherry S., Tropea J.E., Burke T.R., Waugh D.S. (2003). High-resolution structure of the *Yersinia pestis* protein tyrosine phosphatase YopH in complex with a phosphotyrosyl mimetic-containing hexapeptide. Biochemistry.

[172] Sun J.P., Wu L., Fedorov A.A., Almo S.C., Zhang Z.Y. (2003). Crystal structure of the *Yersinia* protein-tyrosine phosphatase YopH complexed with a specific small molecule inhibitor. J. Biol. Chem..

[173] Tautz L., Bruckner S., Sareth S., Alonso A., Bogetz J., Bottini N., Pellecchia M., Mustelin T. (2005). Inhibition of *Yersinia* tyrosine phosphatase by furanyl salicylate compounds. J. Biol. Chem..

[174] Bottini A., Wu B., Barile E., De S.K., Leone M., Pellecchia M. (2016). High-Throughput Screening (HTS) by NMR Guided Identification of Novel Agents Targeting the Protein Docking Domain of YopH. ChemMedChem.

[175] Chen Y.T., Xie J., Seto C.T. (2003). Peptidic alpha-ketocarboxylic acids and sulfonamides as inhibitors of protein tyrosine phosphatases. J. Org. Chem..

[176] Lee K., Boovanahalli S.K., Nam K.Y., Kang S.U., Lee M., Phan J., Wu L., Waugh D.S., Zhang Z.Y., No K.T. (2005). Synthesis of tripeptides as potent *Yersinia* protein tyrosine phosphatase inhibitors. Bioorg. Med. Chem. Lett..

[177] Lee K., Gao Y., Yao Z.J., Phan J., Wu L., Liang J., Waugh D.S., Zhang Z.Y., Burke T.R. (2003). Tripeptide inhibitors of *Yersinia* protein-tyrosine phosphatase. Bioorg. Med. Chem. Lett..

[178] Xie J., Comeau A.B., Seto C.T. (2004). Squaric acids: A new motif for designing inhibitors of protein tyrosine phosphatases. Org. Lett..

[179] Iteman I., Guiyoule A., de Almeida A.M., Guilvout I., Baranton G., Carniel E. (1993). Relationship between loss of pigmentation and deletion of the chromosomal iron-regulated irp2 gene in *Yersinia pestis*: Evidence for separate but related events. Infect. Immun..

[180] Fetherston J.D., Kirillina O., Bobrov A.G., Paulley J.T., Perry R.D. (2010). The yersiniabactin transport system is critical for the pathogenesis of bubonic and pneumonic plague. Infect. Immun..

[181] Perry R.D., Bobrov A.G., Fetherston J.D. (2015). The role of transition metal transporters for iron, zinc, manganese, and copper in the pathogenesis of *Yersinia pestis*. Metallomics.

[182] Stirrett K.L., Ferreras J.A., Jayaprakash V., Sinha B.N., Ren T., Quadri L.E. (2008). Small molecules with structural similarities to siderophores as novel antimicrobials against *Mycobacterium tuberculosis* and *Yersinia pestis*. Bioorg. Med. Chem. Lett..

[183] Ferreras J.A., Gupta A., Amin N.D., Basu A., Sinha B.N., Worgall S., Jayaprakash V., Quadri L.E. (2011). Chemical scaffolds with structural similarities to siderophores of nonribosomal peptide-polyketide origin as novel antimicrobials against *Mycobacterium tuberculosis* and *Yersinia pestis*. Bioorg. Med. Chem. Lett..

[184] Ferreras J.A., Ryu J.S., Di Lello F., Tan D.S., Quadri L.E. (2005). Small-molecule inhibition of siderophore biosynthesis in *Mycobacterium tuberculosis* and *Yersinia pestis*. Nat. Chem. Biol..

[185] Miethke M., Bisseret P., Beckering C.L., Vignard D., Eustache J., Marahiel M.A. (2006). Inhibition of aryl acid adenylation domains involved in bacterial siderophore synthesis. FEBS J..

[186] Bearden S.W., Perry R.D. (1999). The Yfe system of *Yersinia pestis* transports iron and manganese and is required for full virulence of plague. Mol. Microbiol..

[187] Brubaker R.R., Beesley E.D., Surgalla M.J. (1965). *Pasteurella pestis*: Role of pesticin and iron in experiemental plague. Science.

[188] Thomas R., Brooks T. (2004). Common oligosaccharide moieties inhibit the adherence of typical and atypical respiratory pathogens. J Med. Microbiol..

[189] Thomas R., Brooks T. (2006). Attachment of *Yersinia pestis* to human respiratory cell lines is inhibited by certain oligosaccharides. J Med. Microbiol..

[190] Thomas R.J., Hacking A., Brooks T.J. (2007). Structural modification of a base disaccharide alters antiadhesion properties towards *Yersinia pestis*. FEMS Immunol. Med. Microbiol..

[191] Felek S., Tsang T.M., Krukonis E.S. (2010). Three *Yersinia pestis* adhesins facilitate Yop delivery to eukaryotic cells and contribute to plague virulence. Infect. Immun..

[192] Zauberman A., Vagima Y., Tidhar A., Aftalion M., Gur D., Rotem S., Chitlaru T., Levy Y., Mamroud E. (2017). Host Iron Nutritional Immunity Induced by a Live *Yersinia pestis* Vaccine Strain Is Associated with Immediate Protection against Plague. Front. Cell Infect. Microbiol..

[193] Zauberman A., Gur D., Levy Y., Aftalion M., Vagima Y., Tidhar A., Chitlaru T., Mamroud E. (2019). Postexposure Administration of a *Yersinia pestis* Live Vaccine for Potentiation of Second-Line Antibiotic Treatment Against Pneumonic Plague. J. Infect. Dis..

[194] Negus D., Moore C., Baker M., Raghunathan D., Tyson J., Sockett R.E. (2017). Predator Versus Pathogen: How Does Predatory *Bdellovibrio bacteriovorus* Interface with the Challenges of Killing Gram-Negative Pathogens in a Host Setting?. Annu. Rev. Microbiol..

[195] Russo R., Chae R., Mukherjee S., Singleton E.J., Occi J.L., Kadouri D.E., Connell N.D. (2015). Susceptibility of Select Agents to Predation by Predatory Bacteria. Microorganisms.

[196] Russo R., Kolesnikova I., Kim T., Gupta S., Pericleous A., Kadouri D.E., Connell N.D. (2018). Susceptibility of Virulent *Yersinia pestis* Bacteria to Predator Bacteria in the Lungs of Mice. Microorganisms.

[197] Shatzkes K., Chae R., Tang C., Ramirez G.C., Mukherjee S., Tsenova L., Connell N.D., Kadouri D.E. (2015). Examining the safety of respiratory and intravenous inoculation of *Bdellovibrio bacteriovorus* and *Micavibrio aeruginosavorus* in a mouse model. Sci. Rep..

[198] Shatzkes K., Singleton E., Tang C., Zuena M., Shukla S., Gupta S., Dharani S., Rinaggio J., Kadouri D.E., Connell N.D. (2017). Examining the efficacy of intravenous administration of predatory bacteria in rats. Sci. Rep..

[199] Shatzkes K., Tang C., Singleton E., Shukla S., Zuena M., Gupta S., Dharani S., Rinaggio J., Connell N.D., Kadouri D.E. (2017). Effect of predatory bacteria on the gut bacterial microbiota in rats. Sci. Rep..

[200] Atterbury R.J., Hobley L., Till R., Lambert C., Capeness M.J., Lerner T.R., Fenton A.K., Barrow P., Sockett R.E. (2011). Effects of orally administered *Bdellovibrio bacteriovorus* on the well-being and *Salmonella* colonization of young chicks. Appl. Environ. Microbiol..

[201] Willis A.R., Moore C., Mazon-Moya M., Krokowski S., Lambert C., Till R., Mostowy S., Sockett R.E. (2016). Injections of Predatory Bacteria Work Alongside Host Immune Cells to Treat Shigella Infection in Zebrafish Larvae. Curr. Biol..

[202] Findlay J.S., Flick-Smith H.C., Keyser E., Cooper I.A., Williamson E.D., Oyston P.C.F. (2019). Predatory bacteria can protect SKH-1 mice from a lethal plague challenge. Sci. Rep..

[203] Hérelle F. (1917). Sur un microbe invisible antagoniste des bacilles dysentériques. Compte Rendu Académie Sci..

[204] Ravat F., Jault P., Gabard J. (2015). Bactériophages et phagothérapie: Utilisation de virus naturels pour traiter les infections bactériennes. Ann. Burns Fire Disasters.

[205] Filippov A.A., Sergueev K.V., Nikolich M.P. (2012). Can phage effectively treat multidrug-resistant plague?. Bacteriophage.

[206] D’Herelle F. (1925). Essai de traitement de la peste bubonique par le bacteriophage. Presse Med.

[207] Flu P.C. (1929). Antipest bakteriophag und die prophylaxe und therapie der experimentellen pest. Zentbl. Bakteriol. Orig..

[208] Fonquernie I. (1932). Essais de traitement de la peste par le bacteriophage. Bull. Sol. Pathol. Exot..

[209] Lien-Teh W. (1926). A Treatise on Pneumonic Plague.

[210] Naidu B.P.B., Avari G.R. (1932). Bacteriophage in the treatment of plague. Ind. J. Med. Res..

[211] Skurnik M., Strauch E. (2006). Phage therapy: Facts and fiction. Int. J. Med. Microbiol..

[212] Summers W.C. (2001). Bacteriophage therapy. Annu. Rev. Microbiol..

[213] Sulakvelidze A. (2005). Phage therapy: An attractive option for dealing with antibiotic-resistant bacterial infections. Drug Discov. Today.

[214] Filippov A.A., Sergueev K.V., He Y., Huang X.Z., Gnade B.T., Mueller A.J., Fernandez-Prada C.M., Nikolich M.P. (2012). Bacteriophage therapy of experimental bubonic plague in mice. Adv. Exp. Med. Biol..

[215] Balcazar J.L. (2020). Implications of bacteriophages on the acquisition and spread of antibiotic resistance in the environment. Int. Microbiol..

[216] Lu M., Gong T., Zhang A., Tang B., Chen J., Zhang Z., Li Y., Zhou X. (2019). Mobile Genetic Elements in Streptococci. Curr. Issues Mol. Biol..

[217] Patzer S.I., Albrecht R., Braun V., Zeth K. (2012). Structural and mechanistic studies of pesticin, a bacterial homolog of phage lysozymes. J Biol Chem.

[218] Lukacik P., Barnard T.J., Buchanan S.K. (2012). Using a bacteriocin structure to engineer a phage lysin that targets *Yersinia pestis*. Biochem. Soc. Trans..

[219] Lukacik P., Barnard T.J., Keller P.W., Chaturvedi K.S., Seddiki N., Fairman J.W., Noinaj N., Kirby T.L., Henderson J.P., Steven A.C. (2012). Structural engineering of a phage lysin that targets gram-negative pathogens. Proc. Natl. Acad. Sci. USA.

[220] Fetherston J.D., Lillard J.W., Perry R.D. (1995). Analysis of the pesticin receptor from *Yersinia pestis*: Role in iron-deficient growth and possible regulation by its siderophore. J. Bacteriol..

[221] Haffkine W. (1900). Haffkine prohylactic and antipest serum. Public Health Rep..

[222] Yersin A. (1897). Sur la peste bubonique (sérothérapie). Ann. Inst. Pasteur.

[223] Kohler G., Milstein C. (1975). Continuous cultures of fused cells secreting antibody of predefined specificity. Nature.

[224] Zurawski V.R., Haber E., Black P.H. (1978). Production of antibody to tetanus toxoid by continuous human lymphoblastoid cell lines. Science.

[225] Zurawski D.V., McLendon M.K. (2020). Monoclonal Antibodies as an Antibacterial Approach Against Bacterial Pathogens. Antibiotics.

[226] Cowan C., Philipovskiy A.V., Wulff-Strobel C.R., Ye Z., Straley S.C. (2005). Anti-LcrV antibody inhibits delivery of Yops by *Yersinia pestis* KIM5 by directly promoting phagocytosis. Infect. Immun..

[227] Philipovskiy A.V., Cowan C., Wulff-Strobel C.R., Burnett S.H., Kerschen E.J., Cohen D.A., Kaplan A.M., Straley S.C. (2005). Antibody against V antigen prevents Yop-dependent growth of *Yersinia pestis*. Infect. Immun..

[228] Van Blarcom T.J., Sofer-Podesta C., Ang J., Boyer J.L., Crystal R.G., Georgiou G. (2010). Affinity maturation of an anti-V antigen IgG expressed in situ through adenovirus gene delivery confers enhanced protection against *Yersinia pestis* challenge. Gene Ther..

[229] Sittner A., Mechaly A., Vitner E., Aftalion M., Levy Y., Levy H., Mamroud E., Fisher M. (2018). Improved production of monoclonal antibodies against the LcrV antigen of *Yersinia pestis* using FACS-aided hybridoma selection. J. Biol. Methods.

[230] Ivanov M.I., Hill J., Bliska J.B. (2014). Direct neutralization of type III effector translocation by the variable region of a monoclonal antibody to *Yersinia pestis* LcrV. Clin. Vaccine Immunol..

[231] Ivanov M.I., Noel B.L., Rampersaud R., Mena P., Benach J.L., Bliska J.B. (2008). Vaccination of mice with a Yop translocon complex elicits antibodies that are protective against infection with F1- *Yersinia pestis*. Infect. Immun..

[232] Hill J., Copse C., Leary S., Stagg A.J., Williamson E.D., Titball R.W. (2003). Synergistic protection of mice against plague with monoclonal antibodies specific for the F1 and V antigens of *Yersinia pestis*. Infect. Immun..

[233] Hill J., Leary S., Smither S., Best A., Pettersson J., Forsberg A., Lingard B., Lipka A., Brown K.A., Williamson E.D. (2009). N255 is a key residue for recognition by a monoclonal antibody which protects against *Yersinia pestis* infection. Vaccine.

[234] Hill J., Eyles J.E., Elvin S.J., Healey G.D., Lukaszewski R.A., Titball R.W. (2006). Administration of antibody to the lung protects mice against pneumonic plague. Infect. Immun..

[235] Motin V.L., Nakajima R., Smirnov G.B., Brubaker R.R. (1994). Passive immunity to *yersiniae* mediated by anti-recombinant V antigen and protein A-V antigen fusion peptide. Infect. Immun..

[236] Roggenkamp A., Geiger A.M., Leitritz L., Kessler A., Heesemann J. (1997). Passive immunity to infection with *Yersinia* spp. mediated by anti-recombinant V antigen is dependent on polymorphism of V antigen. Infect. Immun..

[237] Une T., Brubaker R.R. (1984). Roles of V antigen in promoting virulence and immunity in *yersiniae*. J. Immunol..

[238] Xiao X., Zhu Z., Dankmeyer J.L., Wormald M.M., Fast R.L., Worsham P.L., Cote C.K., Amemiya K., Dimitrov D.S. (2010). Human anti-plague monoclonal antibodies protect mice from *Yersinia pestis* in a bubonic plague model. PLoS ONE.

[239] Anderson G.W., Worsham P.L., Bolt C.R., Andrews G.P., Welkos S.L., Friedlander A.M., Burans J.P. (1997). Protection of mice from fatal bubonic and pneumonic plague by passive immunization with monoclonal antibodies against the F1 protein of *Yersinia pestis*. Am. J. Trop. Med. Hyg..

[240] Akopyan K., Edgren T., Wang-Edgren H., Rosqvist R., Fahlgren A., Wolf-Watz H., Fallman M. (2011). Translocation of surface-localized effectors in type III secretion. Proc. Natl. Acad. Sci. USA.

[241] Ryzhko I.V., Tsuraeva R.I., Alekseeva L.P., Shcherbaniuk A.I., Mishan’kin M.B. (2003). Use of monoclonal antibodies to plague microbe antigens at the early stage of infection in white mice for enhancement of the late streptomycin and doxycycline treatment. Antibiot. Khimioter..

[242] Daniel C., Dewitte A., Poiret S., Marceau M., Simonet M., Marceau L., Descombes G., Boutillier D., Bennaceur N., Bontemps-Gallo S. (2019). Polymorphism in the *Yersinia* LcrV Antigen Enables Immune Escape From the Protection Conferred by an LcrV-Secreting *Lactococcus lactis* in a Pseudotuberculosis Mouse Model. Front. Immunol..

[243] Daniel C., Sebbane F., Poiret S., Goudercourt D., Dewulf J., Mullet C., Simonet M., Pot B. (2009). Protection against *Yersinia pseudotuberculosis* infection conferred by a *Lactococcus lactis* mucosal delivery vector secreting LcrV. Vaccine.

[244] Silwal P., Paik S., Jeon S.M., Jo E.K. (2020). Nuclear Receptors as Autophagy-Based Antimicrobial Therapeutics. Cells.

[245] Singh B., Varikuti S., Halsey G., Volpedo G., Hamza O.M., Satoskar A.R. (2019). Host-directed therapies for parasitic diseases. Future Med. Chem..

[246] Varma D.M., Zahid M.S.H., Bachelder E.M., Ainslie K.M. (2020). Formulation of host-targeted therapeutics against bacterial infections. Transl. Res..

[247] Wilson C.N., Batra V.K. (2002). Lipopolysaccharide binds to and activates A(1) adenosine receptors on human pulmonary artery endothelial cells. J. Endotoxin Res..

[248] Neely C.F., Jin J., Keith I.M. (1997). A1-adenosine receptor antagonists block endotoxin-induced lung injury. Am. J. Physiol..

[249] Wilson C.N., Vance C.O., Doyle T.M., Brink D.S., Matuschak G.M., Lechner A.J. (2012). A novel post-exposure medical countermeasure L-97-1 improves survival and acute lung injury following intratracheal infection with *Yersinia pestis*. Innate Immun..

[250] Andersson J.A., Fitts E.C., Kirtley M.L., Ponnusamy D., Peniche A.G., Dann S.M., Motin V.L., Chauhan S., Rosenzweig J.A., Sha J. (2016). New Role for FDA-Approved Drugs in Combating Antibiotic-Resistant Bacteria. Antimicrob. Agents Chemother..

[251] Payne F.E., Larson A., Foster W.L., Meyer K.F. (1955). Studies on immunization against plague. IX. The effect of cortisone on mouse resistance to attenuated strains of *Pasteurella pestis*. J. Infect. Dis..

[252] Levy Y., Vagima Y., Tidhar A., Zauberman A., Aftalion M., Gur D., Fogel I., Chitlaru T., Flashner Y., Mamroud E. (2016). Adjunctive Corticosteroid Treatment Against *Yersinia pestis* Improves Bacterial Clearance, Immunopathology, and Survival in the Mouse Model of Bubonic Plague. J. Infect. Dis..

